# Effect of Cyclodextrin Complex Formation on Solubility Changes of Each Drug Due to Intermolecular Interactions between Acidic NSAIDs and Basic H2 Blockers

**DOI:** 10.1021/acs.molpharmaceut.3c00291

**Published:** 2023-09-09

**Authors:** Chihiro Tsunoda, Kanji Hasegawa, Ryosuke Hiroshige, Takahiro Kasai, Hideshi Yokoyama, Satoru Goto

**Affiliations:** Faculty of Pharmaceutical Sciences, Tokyo University of Science, 2641 Yamazaki, Noda, Chiba 278-8510, Japan

**Keywords:** phase solubility diagram, polypharmacy, cyclodextrin, complexation efficiency, NSAIDs

## Abstract

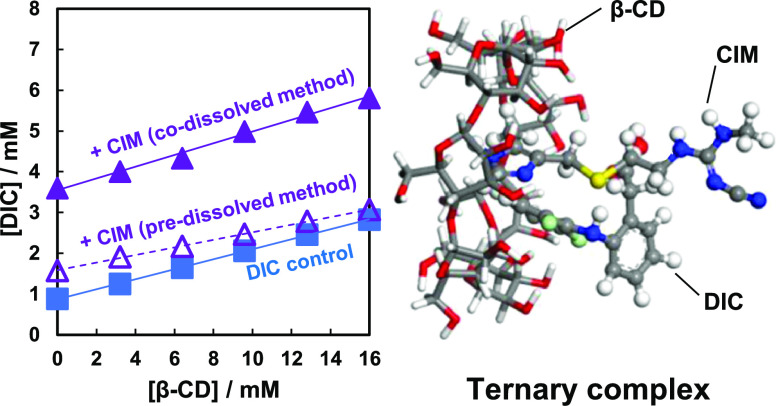

One of the solubilization of poorly water-soluble drugs is the use of cyclodextrin (CD)-based inclusion complexes. On the other hand, few studies have investigated how CD functions on the solubility of drugs in the presence of multiple drugs that interact with each other. In this study, we used indomethacin (IND) and diclofenac (DIC) as acidic drugs, famotidine (FAM) and cimetidine (CIM) as basic drugs, and imidazole (IMZ), histidine (HIS), and arginine (ARG) as compounds structurally similar to basic drugs. We attempted to clarify the effect of β-CD on the solubility change of each drug in the presence of multiple drugs. IND and DIC formed a eutectic mixture in the presence of CIM, IMZ, and ARG, which greatly increased the intrinsic solubility of the drugs as well as their affinity for β-CD. Furthermore, the addition of high concentrations of β-CD to the DIC–FAM combination, which causes a decrease in solubility due to the interaction, improved the solubility of FAM, which was decreased in the presence of DIC. These results indicate that β-CD synergistically improves the solubility of drugs in drug–drug combinations, where the solubility is improved, whereas it effectively improves the dissolution rate of drugs in situations where the solubility is reduced by drug–drug interactions, such as FAM–DIC. This indicates that β-CD can be used to improve the physicochemical properties of drugs, even when they are administered in combination with drugs that interact with each other.

## Introduction

Many active pharmaceutical ingredients (APIs) have low solubility and dissolution rates in aqueous environments such as the gastrointestinal tract.^1^ In fact, an estimated 40% of approved drugs and almost 90% of pipeline drugs in development are composed of poorly water-soluble molecules.^2,3^ Drugs must be present in solution to be absorbed by the body, but the low solubility of poorly water-soluble compounds can result in low drug absorption, leading to increased dosage and the occurrence of side effects due to individual differences in absorption. Therefore, the solubilization of poorly soluble drugs is one of the most important issues in drug formulation. There are various techniques for improving solubility, such as salts, cocrystals, amorphous solid dispersions, and drug nanoparticles, including the use of cyclodextrins (CDs).^4−6^

CD, also called cycloamylose, is a cyclic oligosaccharide consisting of α-1,4-linked glucose units. α-CD with six glucose units, β-CD with seven, and γ-CD with eight are generally known. CDs are hydrophobic on the inside and hydrophilic on the outside, and thus can take in a variety of hydrophobic small molecules in water and form inclusion complexes.^7−13^ This property allows CD to encapsulate highly hydrophobic drugs that are difficult to dissolve in water, thereby improving the solubility and stability of the drugs.^2,7−12,14,15^ In recent years, CD derivatives have also been used to improve the solubility and stability of drugs in Beclery intravenous infusion, a Remdesivir preparation used in the treatment of novel coronaviruses,^16−18^ and the possibilities for CD are unlimited.

In recent years, many attempts have been made to further enhance the solubilizing and stabilizing effects of CD by adding polymers, hyaluronic acid, or amino acids as a third component to prepare a three-component complex in addition to the binary complex of CD as a host molecule and the drug as a guest molecule.^13^ In fact, since prostaglandin (PG) E1 derivative (limaprost), a PG preparation, is chemically very unstable, a three-component complex consisting of limaprost/α-CD/β-CD was developed, and the stability of limaprost was reported to be actually improved.^19^ In addition, as a new approach to the treatment of dry eye disease, a three-component complex consisting of a binary complex of quercetin (QUE) or resveratrol (RSV) and CD, plus hyaluronic acid, has been reported to improve the solubility and stability of QUE or RSV.^20^ Other three-component CD complex crystals containing two different drugs have been reported, and CD complexes with simultaneous release of two drugs are expected to be applied to multiple-drug combination therapy.^21^

With the rapid increase in human life expectancy in recent years, the possibility of developing multiple chronic diseases has increased as survival to old age has increased. In fact, the presence of two or more diseases reaches approximately 40% of individuals over the age of 65 in Europe,^22^ and this prevalence increases further with age. Against this background, there are a large number of patients who use multiple APIs simultaneously, and CD is expected to achieve improved bioavailability by improving drug solubility in the presence of multiple coexisting APIs. However, there are not many reports at this stage on how CD functions in combinations of drugs that interact with each other and alter the physicochemical properties of the drug.

With the rapid aging of the population in the developed world in recent years, it is very important to investigate the characteristics of CD in an environment where multiple drugs interacting with each other coexist in order to expand the use of CD more effectively in the modern era, when not a few patients fall into conditions such as polypharmacy where various drugs are taken simultaneously.^22−24^

In particular, certain combinations of acidic and basic drugs can cause significant changes in physicochemical properties through the formation of ionic liquid eutectic mixtures. For some time now, we have been studying the combination of drugs that interact with each other, focusing on the combination of acidic and basic drugs. Recent studies have reported that equimolar mixtures of thermally treated acidic drug indomethacin (IND) and basic drug lidocaine (LDC) were transformed into eutectic mixtures, and subsequent cooling resulted in the formation of amorphous mixtures with enhanced water solubility.^25^ It is also known that the water solubility of IND is enhanced by LDC, while that of ibuprofen, an acidic drug similar to IND, is suppressed by LDC.^26^ When the basic drugs cimetidine (CIM) and famotidine (FAM) were added to the acidic drugs IND and diclofenac (DIC), a decrease in solubility was observed only with the combination of DIC and famotidine.^27^ NMR analysis suggests that this phenomenon is due to the formation of ion-pair complexes.^27^ It is known that pH fluctuations in the stomach greatly affect drug absorption.^28^ In our previous study, we reported that changes in the apparent hydrophobicity of drugs due to positive acid–base reactions as well as pH changes affect absorption.^29^

In this study, we investigated the effect of CD on the solubility of IND and DIC as acidic drugs, FAM and CIM as basic drugs, and imidazole (IMZ), histidine (HIS), and arginine (ARG) as model drugs having structural similarity to the basic drugs cimetidine and famotidine. The aim of this study was to clarify the effect of CD on the solubility change of each drug in the presence of multiple drugs. FAM is a thioether compound with a guanidyl group, a thiazole ring, and an aminosulfonyldiamino group, whereas CIM is a thioether compound with a guanidyl group and an imidazole ring. IMZ, HIS, and arginine were used as partial structural models of these nitrogen five-membered rings and guanidyl groups, respectively. Although CIM is not used as a histamine H2 receptor antagonist as frequently as FAM, it has structural commonality with FAM, which makes it the best model for basic drugs. As mentioned above, the combination of DIC–FAM is appropriate as a model for multiple-drug combinations in which the physicochemical properties of each API are altered by drug–drug interactions, since the solubility of FAM is reduced by drug–drug interactions with DIC.^27^ In addition, FAM is frequently prescribed to reduce the gastrointestinal burden of drug administration,^30−32^ and it is often reported that the absorption of other concomitant drugs is greatly affected by the gastric acid suppression effect of FAM.^33^

We considered the combination of the proton pump inhibitor basic drug FAM and DIC, one of the nonsteroidal anti-inflammatory drugs (NSAIDs), to be the most appropriate model for a possible clinical drug combination, since gastric protectors are often prescribed together with NSAIDs, since long-term NSAIDs administration can lead to peptic ulcer development.^34^ In addition, the two-component complex of CIM–IND is known to cause an intermolecular interaction between the protons of the imidazole ring of CIM and CO, a part of COOH in IND, forming an amorphous system of the drug and increasing the solubility of IND.^35^ In this context, the IND–CIM combination is also appropriate as a combination of drugs whose solubility is enhanced by the interaction. Therefore, we investigated the effect of CD on the solubility change of drugs in the presence of multiple drugs using IND and DIC as acidic drugs and FAM CIM IMZ, HIS, and ARG as basic drugs.

## Materials and Methods

### Materials

IND, CIM IMZ, HIS, ARG, and β-CD were purchased from FUJIFILM Wako Pure Chemical Corporation (Osaka, Japan); DIC and FAM were purchased from Tokyo Kasei Kogyo Co. The other reagents used were the finest that were commercially available.

### Dissolution Rate Studies

An excess amount of IND, DIC, FAM, and CIM powders, each alone or in two types, were added to 5 mL of 100 mM phosphate buffer (pH 6.5), and the prepared samples were shaken in a Water Bath Shaker PERSONAL-11 (TAITEC Corp. Saitama, Japan) at 120 rpm and 298 K for 4, 8, 24, 48, 72, and 120 h. The undissolved drug present in the sample was then filtered through a 0.22 μm poly(tetrafluoroethylene) membrane filter (SLPT1322NL, hydrophilic PTFE 0.22 μm, Hawach Scientific, Shaanxi, China). After dilution of the sample solutions filtered through the poly(tetrafluoroethylene) membrane filter with an equal volume of a mixture of water and acetonitrile, the concentration of the drug in each sample was analyzed by high-performance liquid chromatography-ultraviolet (HPLC-UV) spectroscopy. The HPLC system (Shimadzu Corp., Kyoto, Japan) consisted of an autosampler (SIL-20A), UV–vis detector (SPD-20A), column oven (CTO-10AS-VP), online degasser (DGU20-A3), and an HPLC pump (LC20-AD). The mobile phase contained 25 mM phosphate buffer and HPLC-grade methanol at a volume ratio of 3:7 and was flowed at a rate of 1 mL/min. A C18 reversed-phase column (Capcell Pak, C18 with a particle size of 5 μm in a container scale of 4.6 mm φ × 250 mm, Shiseido, Tokyo, Japan) was used as the stationary phase and maintained at a temperature of 313 K. The samples (10 μL) were injected into an autosampler, and elution was monitored at a wavelength of 320 nm for IND, 275 nm for DIC, 268 nm for FAM, and 212 nm for CIM.

The saturated concentration (*C*_s_) and *kS*, which is the product of the dissolution rate constant *k* and the solid particle surface area *S*, of IND, DIC, FAM, or CIM were calculated using the Noyes–Whitney equation (eq 1).^36−39^ Here, *C* is the concentration of the drug in solution at time *t*, and *C*_0_ is the concentration of the drug in solution at time *t* = 0. Noyes–Whitney equation is one of the most frequently applied mathematical theories to quantify drug dissolution and is a dissolution model based on diffusion-controlled dissolution using Fick’s first law. In this study, the saturation concentrations *C*_s_ and *kS* for each drug were calculated by fitting a set of experimentally determined values to the integral Noyes–Whitney equation presented in eq 1
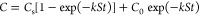
1

### Phase Solubility Studies

Phase solubility diagrams were prepared to determine the ratio of the drug to β-CD in solution and how the solubility of the drug changes when a complex is formed. Excess amounts of the basic drug FAM, CIM, or the acidic drugs IND, DIC, or certain concentrations of CIM, IMZ, HIS, and ARG were added to 5 mL of 100 mM phosphate buffer (pH 6.5) containing 0–16 mM β-CD and samples were placed in a water bath shaker PERSONAL-11 (TAITEC Corp., Saitama, Japan) at 120 rpm and 298 K for 48 or 120 h. In this study, the binary system is defined as when only one drug is added. On the other hand, a three-component system is defined as one in which an acidic drug and a basic drug or IMZ, HIS, or ARG are added one by one, and a total of two drugs are present in the solution. In the codissolved model, the basic drug was added to the acidic sample in a solid state in a test tube containing the acidic drug, followed by the addition of β-CD solution at various concentrations. The phase solubility diagram analysis was performed by adding various concentrations of the β-CD solution. Therefore, in the codissolved model, different drugs contact each other in the solid state before being dissolved. In the predissolved model, 100 mM phosphate buffer (pH 6.5) containing 50 mM IMZ, HIS, and ARG was prepared beforehand, and 0–16 mM β-CD solution was prepared with this solution to prepare the phase solubility diagram. Before the shaken sample solution was diluted, a 0.22 μm poly(tetrafluoroethylene) membrane filter was used to filter the sediment (SLPT 1322 NL, hydrophilic PTFE 0.22 μm, Hawach Scientific, Shaanxi, China). After the sample solution was diluted through a poly(tetrafluoroethylene) membrane filter with an equal volume of a mixture of water and acetonitrile, the concentration of the drug in each sample was analyzed by high-performance liquid chromatography-ultraviolet (HPLC-UV) spectroscopy.

The complex efficiency (CE), which indicates an index of affinity between the drug and CD,^40^ was calculated from the following eq 2 using the slope *a* of each phase solubility diagram
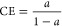
2

### Preparation of the Mixture of Acidic Drug and Basic Drug

To investigate in detail the interaction between acidic drugs and additives in the solid phase, physical mixtures (PM) of acidic drugs and additives were prepared and analyzed by differential scanning calorimetry (DSC) and powder X-ray diffractometry (PXRD). Physical mixtures of acidic and basic drugs were prepared in equimolar proportions. To 0.0800 g of IND, 0.0564, 0.0152, 0.0347, and 0.0390 g of CIM, IMZ, HIS, and ARG were added, respectively, and both were stirred manually with constant pressure using an agate mortar. The physical mixture of DIC and basic drug was prepared in the same way as for IND, adding 0.0852, 0.0230, 0.0524, and 0.0588 g of CIM, IMZ, HIS, and ARG, respectively, to 0.100 g of DIC.

### DSC Measurement

DSC measurements were performed using 5 mg samples in a closed aluminum pan with the Thermo Plus 8230 (Rigaku, Co., Tokyo, Japan) system. These samples were heated from 273 to 425 K at a rate of 10 K/min under a nitrogen gas flow of 30 mL/min.

### PXRD

PXRD pattern measurements were performed using a RINT 2000 (Rigaku Co., Tokyo, Japan) with a Cu Kα radiation source and a Ni filter as the X-ray source, at a voltage of 40 kV and a current of 40 mA. The X-ray irradiation was performed using the parallel-beam method in the 2θ range from 5 to 40° at a scanning velocity of 0.02 steps. The spectra are presented as the average of five scans, and the scanning was conducted in triplicate or a greater number of replicates. To identify the polymorphs of IND, we compared the observed diffractograms of IND single-crystal structures with the published diffractograms. The reproduced diffractogram was calculated from the 3D crystalline structure published by the Reflex Module of Powder Diffraction in Biovia Materials Studio 2020 (Dassault Systems). The 3D crystalline structures of the γ-form (reference code: INDMET) and α-form (reference code: INDMET04) were retrieved from the Cambridge Crystallographic Data Centre (CCDC).

### NMR

The samples for ^1^H NMR and ^13^C NMR measurements were dissolved in methanol-*d*_4_ and D_2_O, respectively. The NMR spectra of the neat drugs and their mixtures at 298 K were recorded on a 400 MHz spectrometer (JNMECZ400, JEOL Ltd., Tokyo, Japan). Spectral analyses were performed using Delta NMR processing software version 5.2.0 (JEOL USA, Inc., Peabody, MA).

### Attenuated Total Reflection-Fourier Transform Infrared (ATR-FTIR) Spectrometry of Mixtures of Basic and Acidic Drugs

The ATR-FTIR spectra were recorded by using an FTIR spectrometer (PerkinElmer Co., Massachusetts, USA) equipped with a universal attenuated total reflectance accessory. The samples were measured over the wavelength range of 400–4000 cm^–1^. A force of 100 N was applied to the sample at the standard temperature. The spectra were the average of 16 scans taken at 1 cm^–1^ resolution.

### Energetics for the Stability of the Acidic Drug/Basic Drug/β-CD Complex in Water

Computational modeling of DIC or IND/FAM or CIM/β-CD complexes in water was performed. Five structures were created for each drug combination (C1: DIC or IND/β-CD complex and isolated FAM or CIM, C2: inclusion of the thiol side of FAM or CIM/β-CD complex and isolated DIC or IND, C3: inclusion of the five-membered ring side of FAM or CIM/β-CD complex and isolated DIC or IND, C4: complexes of FAM or CIM (the thiol side) and DIC or IND in equal proximity to β-CD, C5: complexes of FAM or CIM (the five-membered ring side) and DIC or IND in equal proximity to β-CD). The structures/conformations were geometrically optimized by using the COMPASS III force field for molecular mechanics. Their amorphous cell modules in the periodic structures consisted of the complex embedded in 500 water molecules. The molecular dynamics (MD) processing steps were performed at an interval of 1 fs. In the stabilizing processes for 800 ps, the cell density gradually equilibrated under the NPT (constant number of molecules, stabilized pressure, and stabilized temperature) conditions. Then, the thermal fluctuation motions were sustained to 1000 ps at a temperature of 298 K. The modeling and MD simulations were performed using the Biovia/Accelrys Materials Studio 2022.

## Results and Discussion

### Dissolution Rate Studies

**Figure 1:**
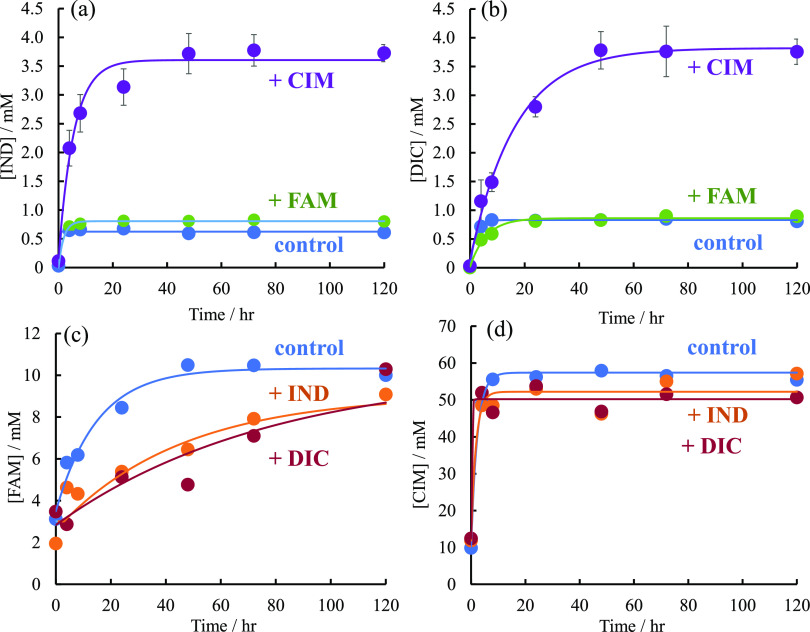
Solubility–time profiles of acidic and basic drugs. Solubility–time profiles of (a) IND in the absence (blue circles) as control and presence of CIM (purple circles) and FAM (green circles), (b) DIC in the absence (blue circles) as control and presence of CIM (purple circles) and FAM (green circles), (c) FAM in the absence (blue circles) as control and presence of IND (orange circles) and DIC (red circles), and (d) CIM in the absence (blue circles) as control and presence of IND (orange circles) and DIC (red circles).

Figure 1 shows the solubility of each drug over time; the saturation concentration *C*_s_ and dissolution rate constant *kS*, calculated from the Noyes–Whitney equation (eq 1), are listed in Table S1. Figure 1a shows the solubility profile of IND, where the saturation solubility *C*_s_ of IND alone was 0.625 mM, whereas that of IND in the presence of CIM was 3.34 mM, indicating that the *C*_s_ of IND increased significantly in the presence of CIM. On the other hand, the dissolution rate constant *kS* was 3.75 h^–1^, the highest value for IND alone, and 0.227 h^–1^, the lowest value for IND in the presence of CIM. Figure 1b shows the solubility profile of DIC. The *C*_s_ of DIC was 0.829 mM in the presence of DIC alone, 0.859 mM in the presence of FAM, and 3.39 mM in the presence of CIM, and as with IND, the solubility of DIC was greatly enhanced in the presence of CIM. The *kS* values of DIC were 0.499, 0.168, and 0.0772 h^–1^ for DIC alone, in the presence of FAM or CIM, respectively. These results of Figure 1a,b indicate that the *C*_s_ of both IND and DIC, which are acidic drugs, is greatly enhanced in the presence of CIM, which is a basic drug. This may be due to the interaction of the carboxylic acids in the molecules of IND and DIC with the imidazole ring in the molecule of CIM, which increases the hydrophilicity of IND and DIC. Figure 1c shows the solubility–time profile of FAM. The values of *C*_s_ in the presence of FAM alone, IND, and DIC were 10.3, 10.3, and 9.08 mM, respectively (Table S1). The values of *kS* in the presence of FAM alone, IND, and DIC were 0.0669, 0.0128, and 0. 0222 h^–1^. The value of *kS* of FAM is greatly reduced in the presence of DIC, which may be a temporary decrease in the solubility of FAM due to the formation of ion pairs between DIC and FAM, as we previously reported. Figure 1d shows the solubility profile of CIM. The *C*_s_ of CIM alone, in the presence of IND, and in the presence of DIC were 57.4, 50.2, and 52.2 mM, indicating that the solubility of CIM with or without the additive did not change significantly.

Proton NMR measurements were performed to investigate in more detail the cause of the increased solubility of acidic drugs in the presence of CIM. Figure S1 shows proton NMR measurements of 20 mM IND, CIM, and an equimolar mixture of IND and CIM. The change in the chemical shifts of IND and CIM alone and in mixtures is that the chemical shifts of the first hydrogen on the imidazole ring of CIM and the seventh hydrogen near the carboxylic acid of IND are very large in the presence of IND and CIM (Figure S2). This suggests that the interaction between the imidazole ring present in the molecule of CIM and the carboxyl group of the acidic drug may improve the solubility of DIC/IND in the presence of CIM and IMZ.

### Dissolution Rate Studies: Phase Solubility Studies of Acidic Drugs IND and DIC

**Figure 2:**
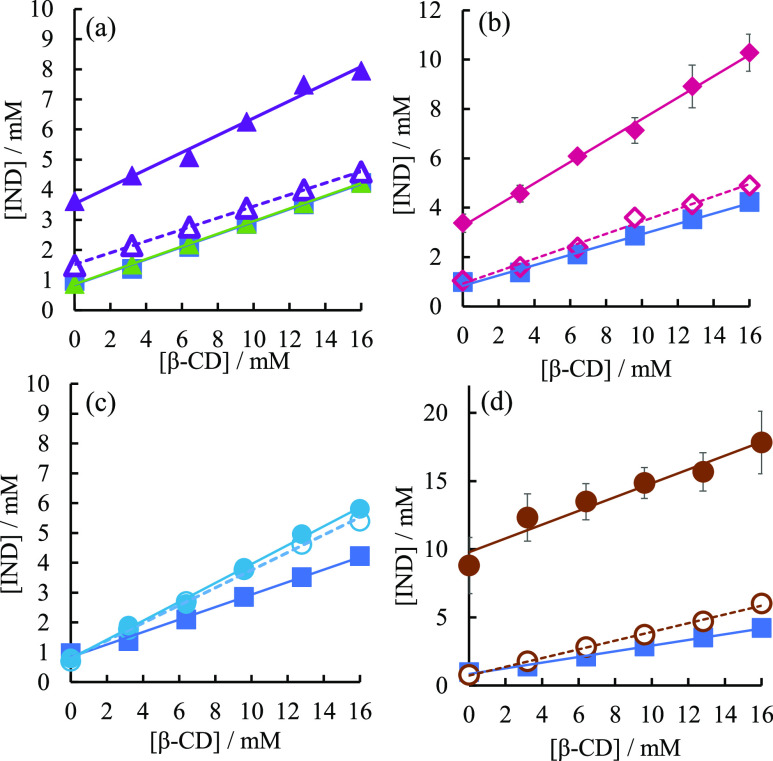
(a) Phase solubility diagram of IND in the absence and presence of CIM and FAM. Without additives (blue square, solid line); in the presence of CIM added by the codissolved method (purple closed triangle, solid line); in the presence of 50 mM CIM added by the predissolved method (purple open triangle, dotted line); in the presence of FAM added by the codissolved method (green closed triangle, solid line). (b) Phase solubility diagram of IND in the absence and presence of IMZ. Without additives (blue squares, solid lines); in the presence of 50 mM IMZ added by the codissolved method (pink closed diamonds, solid lines); in the presence of 50 mM IMZ added by the predissolved method (pink open diamonds, dotted lines). (c) Phase solubility diagram of IND in the absence and presence of HIS. Without additives (blue squares, solid lines); in the presence of 50 mM HIS added by the codissolved method (light blue closed circles, solid lines); in the presence of 50 mM HIS added by the predissolved method (light blue open circles, dotted lines). (d) Phase solubility diagram of IND in the absence and presence of ARG. No additive (blue square, solid line); in the presence of 50 mM ARG added by the codissolved method (brown circle, solid line); in the presence of 50 mM ARG added by the predissolved method (brown open circle, dotted line).

Figure 2a shows the phase solubility diagram of IND with the addition of basic drugs CIM or FAM. When no basic drug was added, the solubility of IND increased in a concentration-dependent manner with β-CD, indicating an A_L_-type phase solubility diagram.^41,42^ When CIM was added by the codissolved method, the solubility of IND increased significantly, regardless of the β-CD concentration. On the other hand, when FAM was added by the codissolved method, the solubility of IND was almost unchanged compared with that of nothing.

Figure 2b shows the phase solubility diagram of IND in the presence of IMZ. When IMZ was added by the codissolved method, as was the case with CIM, the solubility of IND was greatly increased. On the other hand, when IMZ was added by the predissolved method, the solubility of IND was not significantly increased compared with that without any addition. Figure 2c shows the phase solubility diagram of IND in the presence of HIS. HIS showed little difference in the effect of two addition methods, codissolved and predissolved, on the solubility of IND. In addition, the increase in solubility when HIS was added was very small compared to CIM, IMZ, and ARG. Figure 2d shows the phase solubility diagram of IND in the presence of ARG. When added by the codissolved method, ARG improved the solubility of IND the most among the basic drugs used in this study. On the other hand, when ARG was added by the predissolved method, the solubility of IND did not increase much compared with the codissolved method. The stability constant (*K*_c_) of IND in the absence of additives was 315 M^–1^. According to Salústio et al.,^43^ the *K*_c_ of IND for β-CD was 366 M^–1^ at pH 6. Since IND is an acidic drug, the *K*_c_ decreases as the pH increases. Considering this, the *K*_c_ obtained in this experiment was in agreement with the results of previous research.

**Figure 3:**
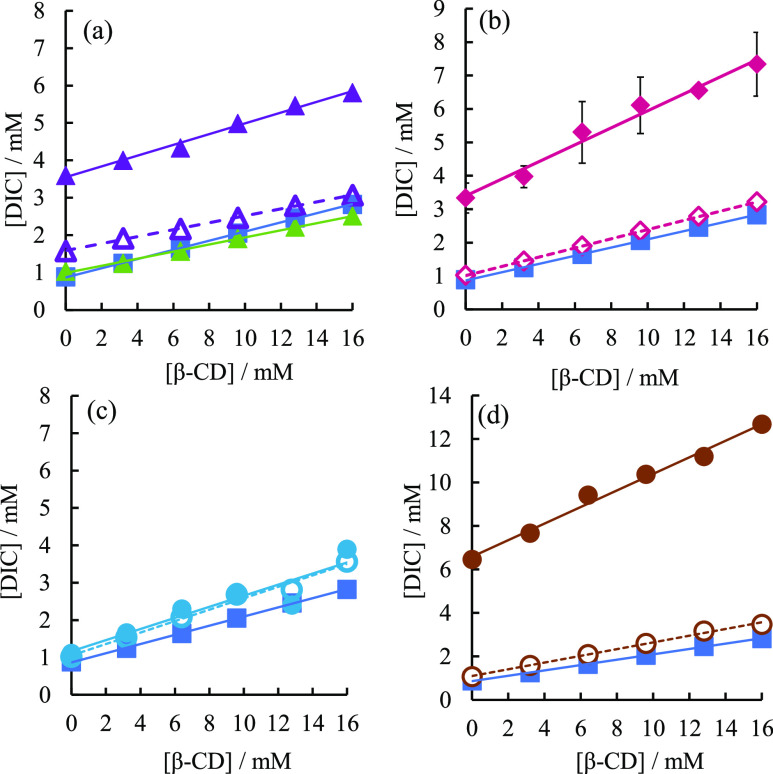
(a) Phase solubility diagram of DIC in the absence and presence of CIM and FAM. Without additives (blue square, solid line); in the presence of CIM added by the codissolved method (purple closed triangle, solid line); in the presence of 50 mM CIM added by the predissolved method (purple open triangle, dotted line); in the presence of FAM added by the codissolved method (green closed triangle, solid line). (b) Phase solubility diagram of DIC in the absence and presence of IMZ. Without additives (blue squares, solid lines); in the presence of 50 mM IMZ added by the codissolved method (pink closed diamonds, solid lines); in the presence of 50 mM IMZ added by the predissolved method (pink open diamonds, dotted lines). (c) Phase solubility diagram of DIC in the absence and presence of HIS. Without additives (blue squares, solid lines); in the presence of 50 mM HIS added by the codissolved method (light blue closed circles, solid lines); in the presence of 50 mM HIS added by the predissolved method (light blue open circles, dotted lines). (d) Phase solubility diagram of DIC in the absence and presence of ARG. No additive (blue square, solid line); in the presence of 50 mM ARG added by the codissolved method (brown circle, solid line); in the presence of 50 mM ARG added by the predissolved method (brown open circle, dotted line).

The phase solubility diagram of DIC in the presence of the basic drugs CIM and FAM, and that of DIC alone, shows an A_L_-type phase solubility diagram in which DIC increases linearly with β-CD concentration (Figure 3a). In the A_L_-type, the stoichiometry of the complex does not change and a 1:1 complex is always formed.^41,42^ This indicates that DIC forms a 1:1 soluble complex with β-CD in solution. Similar to IND, the solubility of DIC was greatly enhanced when CIM was added by the codissolved method, regardless of the β-CD concentration. On the other hand, the solubility of DIC did not improve much when CIM was added by the predissolved method. When FAM was added by the codissolved method, the solubility of DIC in the presence of 16 mM β-CD was decreased. Figure 3b is the phase solubility diagram of DIC in the presence of IMZ. When IMZ was added by the codissolved method, the solubility of DIC increased significantly, similar to when CIM was added. On the other hand, when IMZ was added by the predissolved method, the solubility of DIC did not increase significantly compared to when nothing was added (Figure 3b). Figure 3c is the phase solubility diagram of DIC in the presence of HIS. Similar to IND, there was no significant difference in the solubility of DIC when HIS was added by the codissolved method and the predissolved method. Also, the increase in solubility with the addition of HIS was very small compared to those of CIM, IMZ, and ARG. Figure 3d is the phase solubility diagram of DIC in the presence of ARG. Similar to IND, the addition of ARG by the codissolved method significantly increased the solubility of DIC. On the other hand, the addition of ARG by the predissolved method increased the solubility of DIC, but the solubility of DIC did not increase significantly compared to the addition by the codissolved method (Figure 3d). The *K*_c_ value of DIC in the absence of additives was 161 M^–1^. According to M.L. Manca et al.,^44^ the *K*_c_ of DIC for β-CD is 159 M^–1^ at pH 7.0, and the *K*_c_ of DIC in the absence of additives obtained in this experiment agrees with the results of previous research.

**Figure 4:**
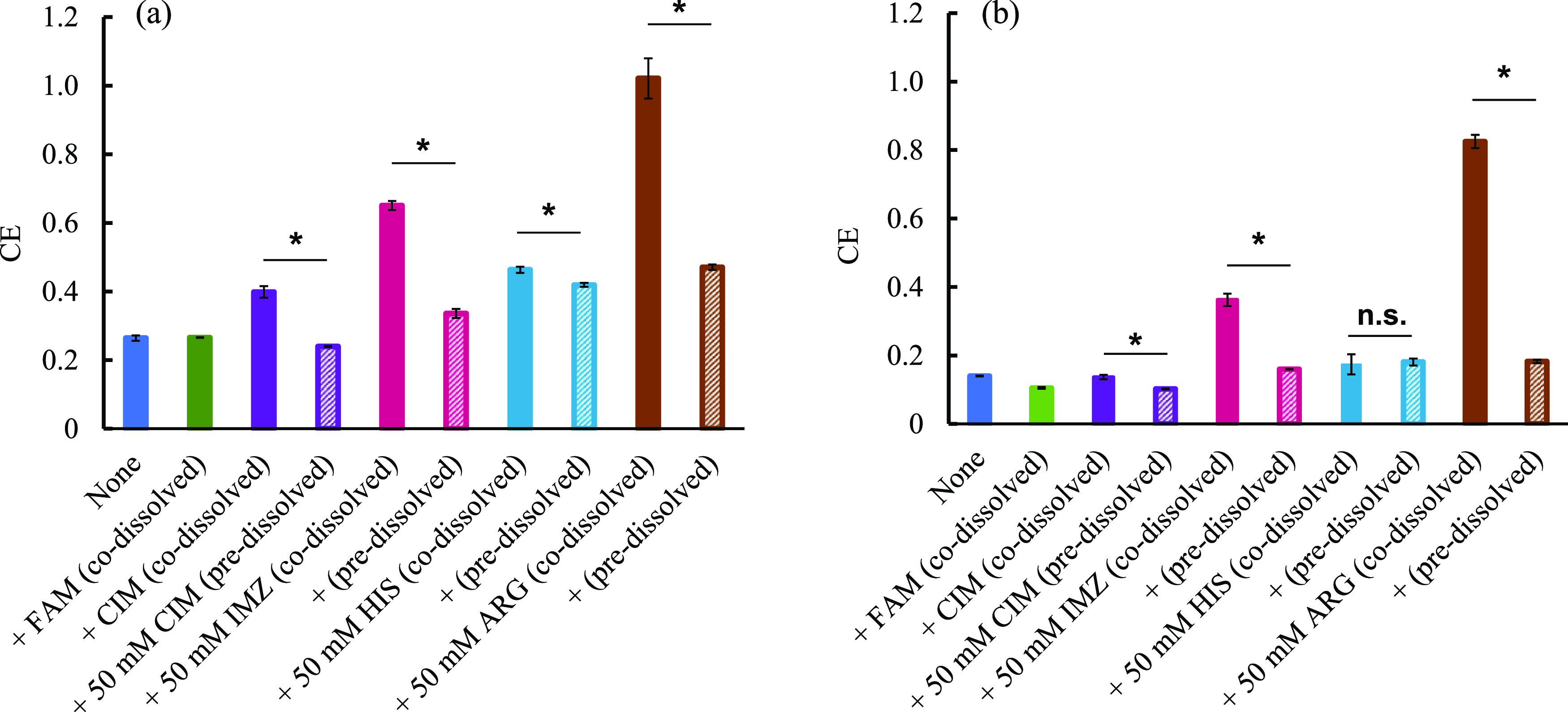
(a) Complexation efficiency CE of IND in the presence of various basic drugs. (b) Complexation efficiency CE of DIC in the presence of various basic drugs. Values are the mean ± SE; *p < 0.05, n.s.: not significant by Student’s *t* test.

Next, the values of the complexation efficiency (CE) of IND and DIC with β-CD in the presence of various additives are discussed. The complexation efficiencies of IND and β-CD in the presence of each additive are shown in Figure 4a. In this study, the intrinsic solubility of IND or DIC (*S*_0_) is greatly changed by the additives. Therefore, we evaluated the difference in affinity between acidic drugs and β-CD using complexation efficiency (CE) rather than the stability constant including *S*_0_ as a parameter in the calculation. First, the value of the CE for IND in the presence of each additive is shown in Figure 4a. With no addition (None), the complexation efficiency of IND was 0.2645. On the other hand, the complexation efficiency between IND and β-CD was 0.3910 (codissolved method) or 0.2399 (predissolved) in the presence of CIM, 0.6507 (codissolved) or 0.3360 (predissolved) in the presence of IMZ, 0.4635 (codissolved) or 0.4195 (predissolved) in the presence of HIS, and 3.860 (codissolved) or 0.4708 (predissolved) in the presence of ARG. As shown in these results, the affinity between IND and β-CD was significantly increased when CIM, IMZ, and ARG were added in the solid state by the codissolved method. The CE values indicate that IMZ, HIS, and ARG increase the affinity of IND and β-CD regardless of the addition method.

The value of CE for DIC was 0.140 in the absence of any additive (none), 0.137 (codissolved), 0.102 (predissolved) in the presence of CIM, 0.363 (codissolved) or 0.160 (predissolved), 0.174 (codissolved) or 0.181 (predissolved) in the presence of HIS, and 0.826 (codissolved) or 0.183 (predissolved) in the presence of ARG (Figure 4b). As can be seen from these results, the affinities of IND and β-CD were found to increase significantly, especially when ARG was added in the solid state by the codissolved method. Unlike IND, the CE of DIC did not increase when CIM was added in the solid state by the codissolved method. As can be seen from Figure 2, some additives have different effects on the solubility of IND depending on the method of addition, while others have almost the same effect on the solubility of IND regardless of the method of addition. For example, CIM, IMZ, and ARG effectively improved the solubility of IND when they were added by the codissolved method rather than by the predissolved method, whereas in HIS, there is no significant difference in the solubility of IND between the codissolved method and the predissolved method. This suggests that CIM and IMZ, which showed a greater increase in the solubility of IND in the codissolved method than in the predissolved method, showed higher solubility in the codissolved method than in the predissolved method due to the formation of a eutectic mixture and the lowering of melting point caused by the direct contact of additive IND in the solid state during sample preparation. For these reasons, we decided to prepare a physical mixture of acidic drugs with CIM, IMZ, HIS, and ARG and investigate their physicochemical properties with DSC and PXRD.

### Phase Solubility Studies of Basic FAM and CIM

**Figure 5:**
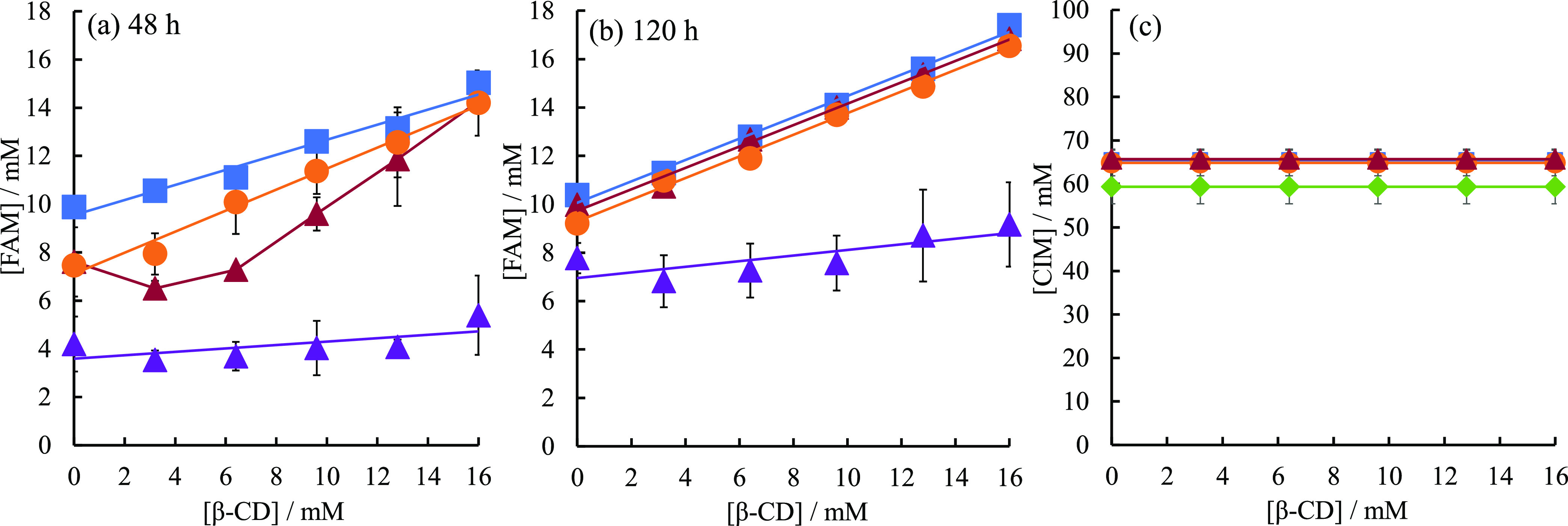
(a) Phase solubility diagram of FAM in the absence and presence of IND and DIC after 48 h of shaking. (b) Phase solubility diagram of FAM in the absence and presence of IND and DIC after 120 h of shaking. (c) Phase solubility diagram of CIM in the absence and presence of IND and DIC after 120 h of shaking. Without additives (blue squares); in the presence of IND added by the codissolved method (orange circles); in the presence of DIC added by the codissolved method (rouge triangles); in the CIM added by the codissolved method (purple triangles); in the FAM added by the codissolved method (green diamonds).

The phase solubility diagram of FAM after shaking at 48 h is shown in Figure 5a. The phase solubility diagram of FAM monotony shows that the solubility of FAM increases in a β-CD concentration-dependent manner, suggesting that FAM forms a one-to-one soluble inclusion complex with β-CD in solution. On the other hand, in the presence of DIC and IND, the solubility of FAM decreased at β-CD concentrations from 0 to 6.4 mM. However, at β-CD concentrations of 9.6–16 mM, the solubility of FAM was improved, and the solubility of FAM was almost the same as that of FAM alone without any addition. Previously, we have reported that NMR measurements in the presence of FAM and DIC showed that the amino group of DIC and the guanidyl group of FAM shifted to higher magnetic fields, suggesting that FAM interacts with DIC in solution.^27^ The solubilities of DIC and FAM are decreased by the complex formation due to the interaction between DIC and FAM, which cancels out the polarization of each other. The phase solubility diagram results showed that high concentrations of β-CD improved the solubility of FAM even in the presence of DIC, suggesting that high concentrations of β-CD decoupled the interaction between FAM and DIC and improved the dissolution rate of each drug. On the other hand, low concentrations of CD conversely decreased the dissolution rate of FAM, indicating a concentration-dependent functional difference of CD for the DIC–FAM interaction. In the presence of CIM, which is a basic drug, the solubility of FAM was greatly reduced and it was found that FAM was not solubilized by β-CD. Figure 5b shows the solubility phase diagram of FAM after 120 h of shaking. 120 h of shaking showed no significant difference from that of FAM alone, either in the presence of DIC or IND. As shown in Figure 1c, the solubility of FAM decreased by half after 48 h of shaking in the presence of IND and DIC, but after 120 h of shaking in the presence of IND or DIC, the solubility of FAM was almost the same as that in the absence of any addition. These results indicate that the addition of IND and DIC decreases the dissolution rate of FAM but does not change the saturation concentration. In the solubility phase diagram after 120 h, it was confirmed that there was no significant difference in the solubility of FAM in the presence of IND and DIC because FAM reached the saturation concentration even in the presence of IND and DIC. On the other hand, in the presence of CIM, the solubility of FAM was still greatly reduced after 120 h of shaking, indicating that the solubilizing effect of β-CD was negligible. FAM was not solubilized by β-CD in the presence of CIM either after 48 or 120 h of shaking, indicating B_I_-type.^39,40^ This suggests that FAM and CIM form aggregates and precipitates. Suppose CIM and FAM are present in the solution as monomolecular, the solubility of the drug is expected to increase in a β-CD concentration-dependent manner due to the solubilizing effect of β-CD. Since FAM is not subject to the solubilization effect by β-CD in the presence of CIM after either 48 or 120 h shaking, it is considered that FAM and CIM form something like aggregates and precipitates. If CIM and FAM are monomolecular and present in solution, it is expected that the solubility of the drug will increase in a concentration-dependent manner of β-CD due to the solubilizing effect of β-CD. The very low stability constant of FAM in the presence of CIM and the very low solubility of FAM at 0 mM β-CD in the phase solubility diagram after 120 h of shaking suggest that FAM and CIM formed insoluble aggregates and individual drug molecules could not enter the hydrophobic cavities inside β-CD. According to the Henderson–Hasselbarch equation, FAM (p*K*_a_ 7.06) and CIM (p*K*_a_ 7.05), which are basic drugs, become less soluble as the pH increases. As the concentration of one basic drug increases, the pH of the solution increases, and the other drug becomes less soluble. Figure 5c shows the phase solubility diagram of CIM, where the solubility of CIM is constant regardless of the concentration of β-CD, indicating that CIM is not affected by the solubilization effect of β-CD. On the other hand, as shown in Figure S4, the solubility of CIM increased in a concentration-dependent manner with β-CD in buffer solutions at pH 7.5 and 8.5, and the stability constants of CIM and β-CD were 35.79 M^–1^ at pH 7.5 and 64.4 M^–1^ at pH 8.5, indicating that CIM is a basic drug. The stability constants of CIM and β-CD increased at higher pH, where the ratio of the basic drug CIM to molecular type increased. In order to clarify the reason CIM is not at all solubilized by β-CD in the buffer solution at pH 6.5, we analyzed the change in the phase solubility diagram of CIM with the pH change using the Henderson–Hasselbarch equation. The total concentration of CIM ([CIM]_t_) was set as the sum of CIM in molecular form, CIM in ionic form, and CIM/β-CD. Since CIM is a basic drug, we derived eq S3 as [CIM]_ion_/[CIM]_mol_ = 10^p*K*_a_-pH^ (see eq S3). [CIM]*_t_* in eq S3 is expressed by substituting 16 mM for the β-CD concentration and the stability constant *K* for the stability constant value obtained from the phase solubility diagram at each pH. Then, the data obtained by the nonlinear least-squares method were calculated using the solver module of Microsoft Excel so that the residual least-squares sum between the total CIM concentration expressed by the theoretical formula of eq S3 and the CIM concentration actually measured under each condition was minimized. After fitting, the CIM concentration [CIM]_mol_ in molecular form was calculated. Based on the calculated molecular CIM concentration [CIM]_mol_, the ionic CIM concentration [CIM]_ion_ was calculated using eq S2. As a result, the concentrations of CIM in molecular form and CIM in ionic form at each pH in the presence of 16 mM β-CD are shown in Figure S4. As shown in Figure S5, the calculated proportion of ionic CIM is very high at pH 6.5. This suggests that CIM was not solubilized by β-CD in the buffer solution at pH 6.5 because the proportion of CIM in the ionic form, which is highly hydrophilic, was high, and CIM could not enter the hydrophobic cavity inside the CD. The solubility of CIM remained constant regardless of the β-CD concentration even in the presence of acidic IND and DIC.

Molecular dynamics calculations were performed to realize the molecular size (Figures S6 and S7). Figure S6a shows the molecular dynamic trajectory of neutral DIC/FAM/β-CD complex. After 800 ps, the composite was completely equilibrated. In addition, the energy level did not switch and C4 was the most stable. Figure S7a shows snapshots of the most stable structures of the DIC/FAM/β-CD complex. This suggests that the ternary complexes were stable in DIC/FAM/β-CD complexes. In the DIC/FAM/β-CD and DIC/CIM/β-CD complexes, ternary complexes C4 and C5 were the most stable, respectively (Figure S6a,b). In the IND/FAM/β-CD and IND/CIM/β-CD complexes, binary complexes C1 and C2 were the most stable, respectively (Figure S6c,d). In this modeling, we do not think that theoretical results could be obtained by statistical thermodynamic calculations, but only to confirm whether the size of the β-CD inclusion complex is appropriate.

### Interactions between Acidic and Basic Drugs in Solid Phase

**Figure 6:**
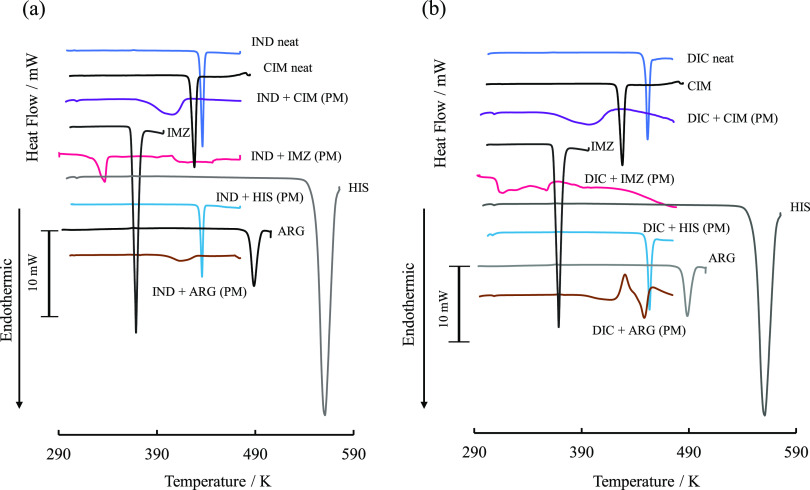
(a) DSC thermograms of a physical mixture consisting of IND and basic drugs. (b) DSC thermograms of a physical mixture consisting of DIC and basic drugs.

**Figure 7:**
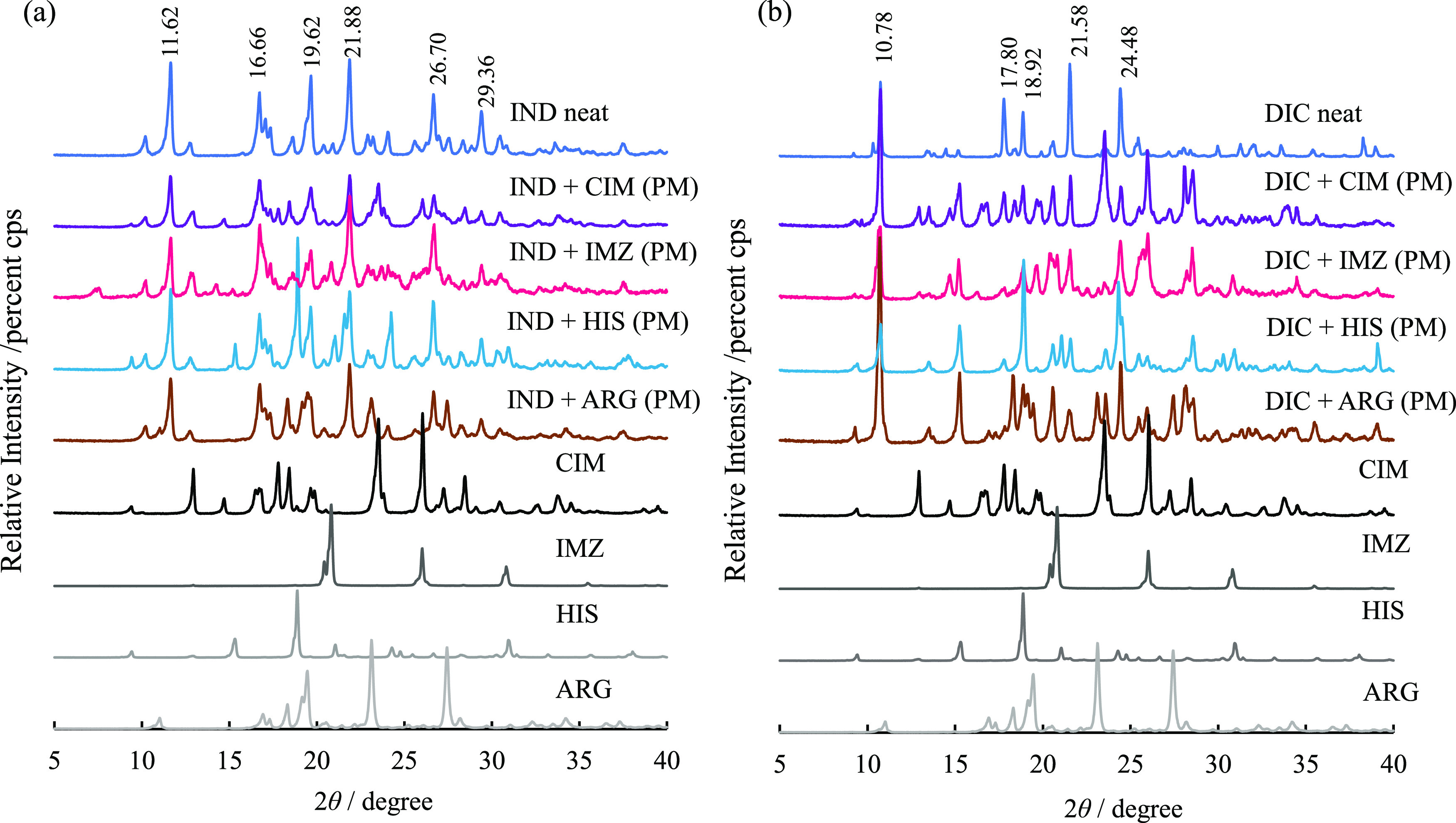
(a) Diffractograms of an equimolar physical mixture of IND and a basic drug. (b) Diffractogram of an equimolar physical mixture of DIC and a basic drug.

Since the phase solubility diagram of acidic drugs showed significant differences in the solubility of acidic drugs depending on the addition method of basic drugs, the influence of physicochemical interactions between acidic and basic drugs in the solid phase was investigated by thermal analysis. DSC thermograms of equimolar mixtures of acidic and basic drugs prepared with PM were obtained (Figure 6). In the following text, equimolar physical mixtures of acidic and basic drugs are denoted by acidic/basic drugs. The melting points of IND and CIM were 433.1 and 413.4 K, respectively, whereas the melting point of the mixture was 379.1 K, which was significantly lower. The melting points of IND/IMZ and IND/ARG were also significantly lower than those of IND/CIM. On the other hand, the melting point of IND/HIS was 433.2 K, indicating that IND and HIS do not interact in the solid phase. The solubility of IND increased significantly in the codissolved method when CIM, IMZ, and ARG were added to the phase solubility diagram of IND in Figure 2, whereas the solubility of IND did not increase much in the predissolved method. The codissolved method significantly increased the solubility of IND, whereas the predissolved method did not increase the solubility of IND as much as the codissolved method did. In contrast to other additives, when HIS was added, there was no difference in CE values between the predissolved method and the codissolved method. This is because IND and HIS did not interact in the solid phase; that is, they did not form a eutectic mixture. The melting point of DIC alone was 449.3 K, corresponding to Form II;^45^ as in IND, in equimolar mixtures of DIC with CIM prepared by the PM method, the melting peak of DIC in the mixture broadened and shifted to a lower temperature, 397 K. On the other hand, the melting point of DIC in DIC/HIS was 453.3 K, almost the same as that of neat DIC. This suggests that, as with IND, when CIM, IMZ, and ARG were added to the phase solubility diagram of DIC in Figure 3b, the codissolved method significantly increased the solubility of IND, whereas the predissolved method did not significantly increase the solubility of IND because in the codissolved method, solid acidic and basic drugs coexisted and interacted with each other during sample preparation, forming a eutectic mixture. The FTIR spectra of the PMs of basic and acidic drugs shown in Figure S8 indicate that the bands around 1680–1700 cm^–1^ due to the intramolecular carboxylic acids of IND and DIC are greatly reduced in the PMs with IMZ and ARG. The formation of these comelt mixtures may be caused by the interaction between the intramolecular carboxylic acids of IND and DIC, which are acidic drugs, and the amines present in the guanidyl groups of ARG. The PM composed of CIM and IND showed no significant change in the band around 1680–1700 cm^–1^, which is due to intramolecular carboxylic acids of IND, while the PM composed of CIM and DIC exhibited a decrease in the band specific to carboxylic acids (Figure S8). Figure 7 shows the diffractograms of the physical mixtures of IND and basic drugs and of DIC and basic drugs measured by PXRD. As shown in Figure 7, neat IND exhibits 2θ = 11.62, 16.66, 19.62, 21.88, 26.70, and 29.36° characteristic high-intensity diffraction peaks, indicating that it is a γ-form IND (Figure S9). In IND/CIM, the peaks at 2θ = 19.62, 21.88, 26.70, and 29.36° of IND were significantly reduced; in IND/IMZ, the peaks at 2θ = 11.62, 19.62, 21.88, 26.70, and 29.36° were significantly reduced. However, no new diffraction patterns appeared in IND/CIM and IND/IMZ. Neat DIC showed characteristic intense diffraction patterns at 2θ = 10.78, 17.80, 18.92, 21.58, and 24.48°. CIM and DIC/IMZ, the diffraction intensities, were significantly reduced. On the other hand, as in IND, no new peaks different from those of the parent component appear in the equimolar physical mixture of DIC and the basic drug.

## Conclusions

β-CD synergistically enhances the solubility of drugs in drug–drug combinations where the solubility is enhanced by the formation of a mixture, whereas it effectively enhances the dissolution rate of drugs in situations where the drug–drug interaction, such as DIC–FAM, reduces the solubility. It is considered that the drugs did not receive the solubilizing effect of β-CD in the presence of FAM and CIM, which are basic drugs because they aggregate with each other. Therefore, solubilizers other than β-CD may be useful for improving the solubility of drugs that cause aggregation in the presence of other drugs. These results suggest that intermolecular interactions may be responsible for the enhanced solubility of drugs. These results provide new insights into the function of CDs in altering drug solubility through intermolecular interactions and are expected to provide important clues for improving the bioavailability of individual drugs under multiple-drug combination conditions.

## Supporting Information Available

Saturation concentration (*C*_s_) and dissolution rate constant (*kS*) for each acidic and basic drug in the presence of various additives calculated from the Noyes–Whitney equation (eq 1) (Table S1); solution ^1^H NMR spectra of 20 mM CIM, 20 mM IND, and 20 mM IND/CIM in methanol-*d*_4_ (Figure S1); change in chemical shift between IND alone and IND in IND/CIM (a) and between CIM alone and IND in IND/CIM (b) (Figure S2); solution-state ^13^C NMR spectra of 5 mM DICNa, 5 mM DICNa + 15 mM ARG, and 15 mM ARG in D_2_O (Figure S3); experimental ^13^C NMR chemical shifts for (a) 5 mM DICNa, (b) 5 mM DICNa + 15 mM ARG, and (c) 15 mM ARG (Table S2); phase solubility diagram of CIM (Figure S4); concentrations of CIM in ionic form, CIM in molecular form, and CIM/β-CD in the presence of 16 mM β-CD in the phase solubility diagram of CIM at each pH calculated by the solver module of Microsoft Excel (Figure S5); molecular dynamic trajectory of neutral (a) DIC/FAM/β-CD complex, (b) DIC/CIM/β-CD complex, (c) IND/FAM/β-CD complex, and (d) IND/CIM/β-CD complex: C1: DIC or IND/β-CD complex and isolated FAM or CIM, C2: inclusion of the thiol side of FAM or CIM/β-CD complex and isolated DIC or IND, C3: inclusion of the five-membered ring side of FAM or CIM/β-CD complex and isolated DIC or IND, C4: complexes of FAM or CIM (the thiol side) and DIC or IND in equal proximity to β-CD, C5: complexes of FAM or CIM (the five-membered ring side) and DIC or IND in equal proximity to β-CD (Figure S6); snapshots for the most stable structures of (a) DIC/FAM/β-CD complex, (b) DIC/CIM/β-CD complex, (c) IND/FAM/β-CD complex, and (d) IND/CIM/β-CD complex in C1–C5 (Figure S7); FTIR spectra of physical mixtures (PM) of basic and acidic drugs (Figure S8); and diffractograms of γ-form and α-form IND by actual measurement and those reported in the literature (Figure S9). The data can be obtained free of charge from The Cambridge Crystallographic Data Centre (CCDC) via www.ccdc.cam.ac.uk/getstructures (PDF)

## Abbreviations

APIs: active pharmaceutical ingredients
NSAIDs: nonsteroidal anti-inflammatory drugs
LDC: lidocaine
IND: indomethacin
DIC: diclofenac
FAM: famotidine
CIM: cimetidine
CD: cyclodextrin
IMZ: imidazole
HIS: histidine
ARG: l-arginine
PG: prostaglandin
QUE: quercetin
RSV: resveratrol
PM: physical mixture
CE: complexation efficiency
HPLC-UV: high-performance liquid chromatography-ultraviolet
DSC: differential scanning calorimetry
PXRD: powder X-ray diffractometer
ATR-FTIR: attenuated total reflection-Fourier transform infrared
MD: molecular dynamics
