# Supplementary material for: Effect of Cyclodextrin Complex Formation on Solubility Changes of Each Drug Due to Intermolecular Interactions between Acidic NSAIDs and Basic H2 Blockers
Source: Mol Pharm. 2023 Sep 9;20(10):5032–42. doi: 10.1021/acs.molpharmaceut.3c00291 (PMC10548472; doi:10.1021/acs.molpharmaceut.3c00291)
Supplement: Supplementary file 1 — mp3c00291_si_001.pdf [file mp3c00291_si_001.pdf]

Effect of cyclodextrin complex formation on solubility changes of each drug due to intermolecular interactions between acidic NSAIDs and basic H2 blockers

Chihiro Tsunoda, Kanji Hasegawa, Ryosuke Hiroshige, Takahiro Kasai, Hideshi Yokoyama, Satoru Goto*

Faculty of Pharmaceutical Sciences, Tokyo University of Science

2641 Yamazaki, Noda, Chiba, Japan 278-0037

*Corresponding author: s.510@rs.tus.ac.jp

Supporting Information

Table S1 Saturation concentration C_s and dissolution rate constant *k*S for each acidic and basic drug in the presence of various additives calculated from the Noyes-Whitney equation (eq1).

	Cs / mM	k S / hr^{-1}
IND	0.625	3.75
IND in the presence of FAM	0.807	0.522
IND in the presence of CIM	3.34	0.227
DIC	0.829	0.499
DIC in the presence of FAM	0.859	0.168
DIC in the presence of CIM	3.39	0.0772
FAM	10.3	0.0669
FAM in the presence of DIC	10.3	0.0128
FAM in the presence of IND	9.08	0.0222
CIM	57.4	0.433
CIM in the presence of DIC	50.2	4.46
CIM in the presence of IND	52.2	0.554

Fig. S1

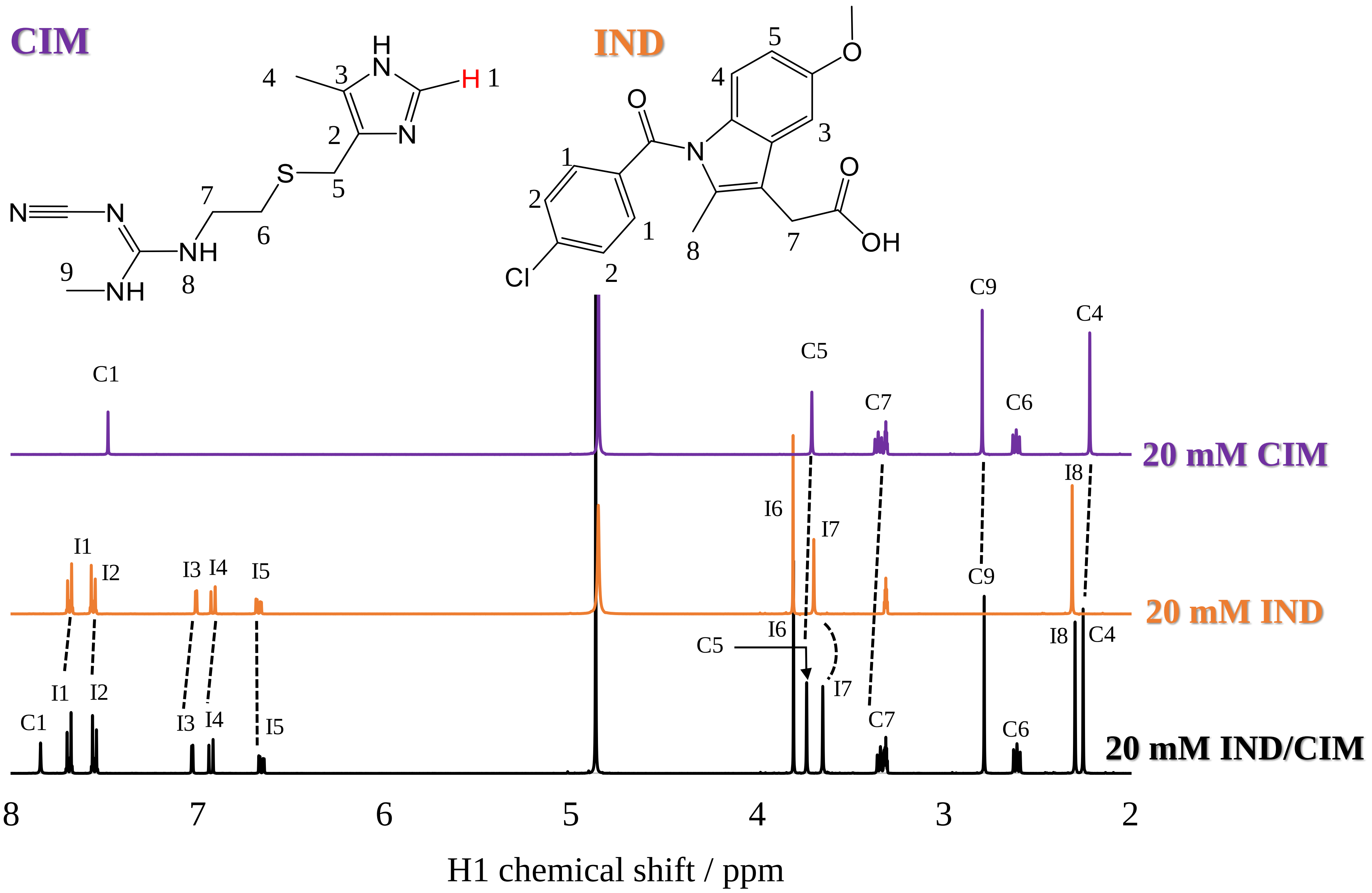

Figure S1. (a) Solution ^{1}H NMR spectra of 20 mM CIM, 20 mM IND, 20 mM IND/CIM in methanol-d_4.

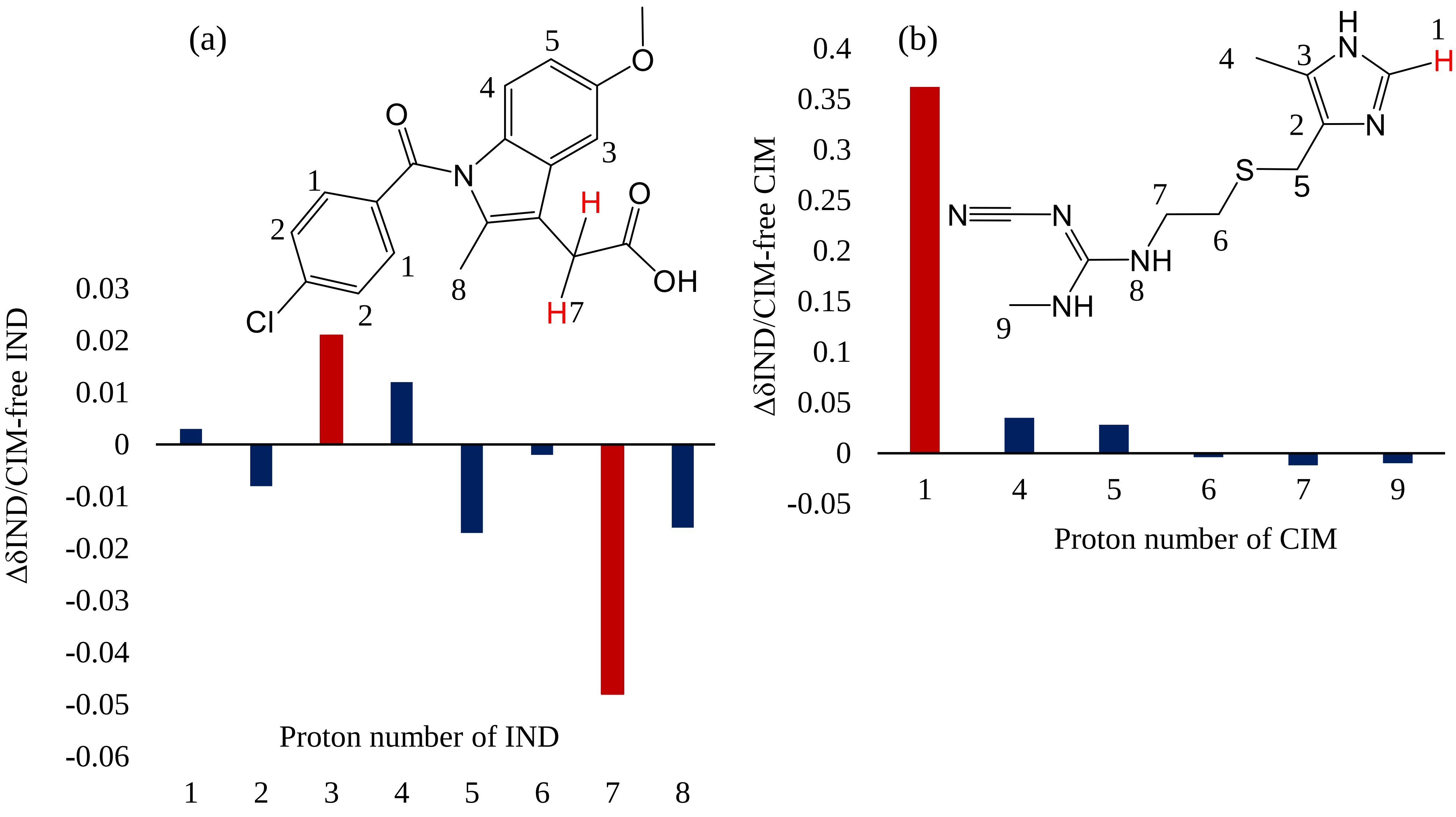

Figure S2. (a) Change in chemical shift between IND alone and IND in IND/CIM. (a) Change in chemical shift between CIM alone and IND in IND/CIM.

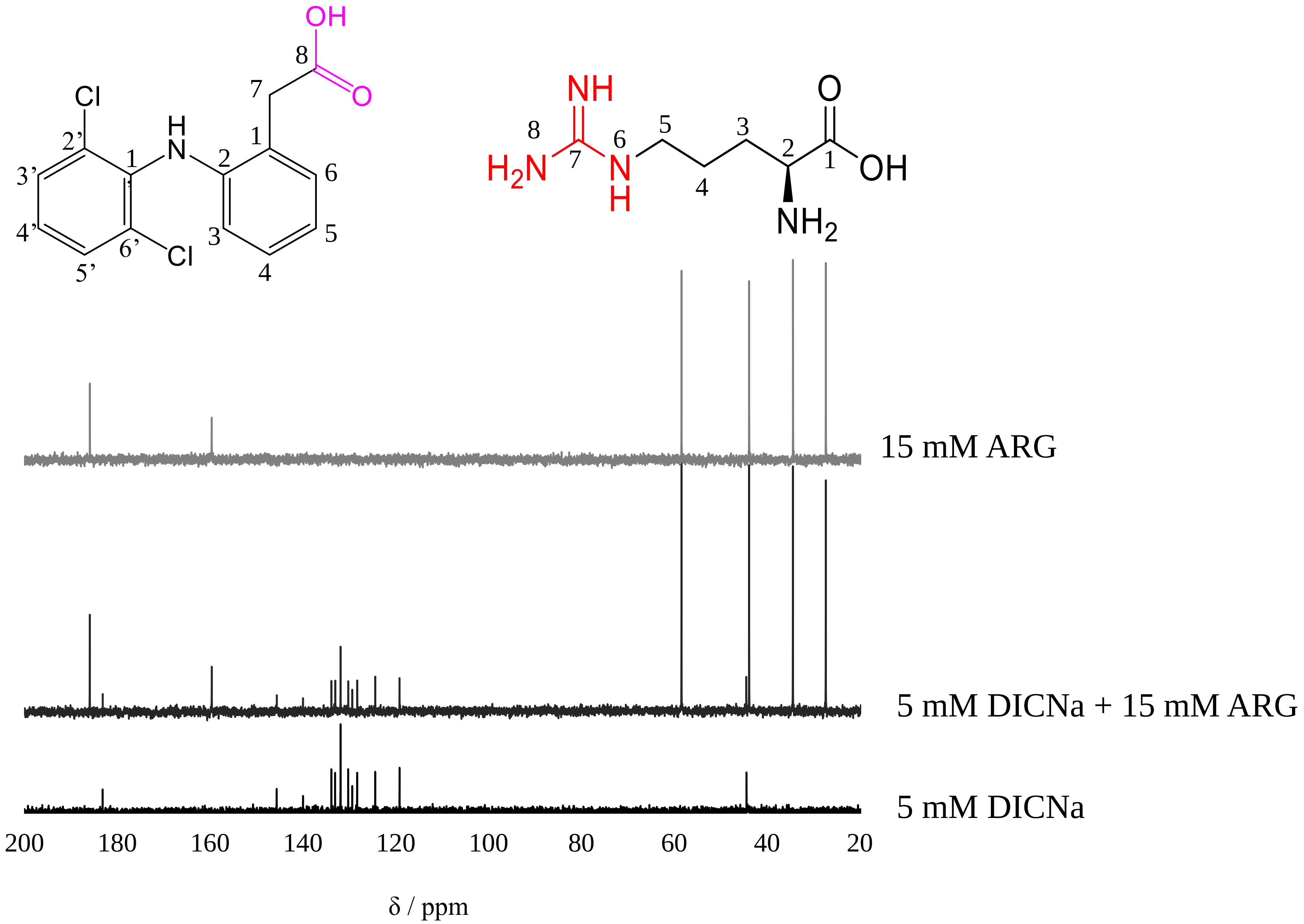

Figure S3. The solution-state ^{13}C-NMR spectra of 5mM DICNa, 5mM DICNa + 15 mM ARG and 15 mM ARG in D_2O.

Table S2. The experimental ^{13}C-NMR chemical shifts for (a) 5 mM DICNa (b) 5 mM DICNa + 15 mM ARG (c) 15 mM ARG.

	Carbon No.	(a) 5 mM DICNa	(b) 5mM DICNa + 15 mM ARG	(c) 15mM ARG
DICNa	8	183.117	183.107	
	1	145.62	145.6	
	2	139.956	139.956	
	6	133.842	133.833	
	1'	133.037	132.99	
	3', 5'	131.859	131.868	
	4	130.22	130.211	
	2'	129.348	129.348	
	4'	128.294	128.256	
	5	124.394	124.394	
	3	119.152	119.162	
	7	44.407	44.435	
ARG	1		185.896	185.867
	7		159.62	159.63
	2		58.407	58.397
	5		43.832	43.832
	3		34.402	34.373
	4		27.311	27.311

For basic drugs, the relationship between pH and pKa of the drug is as follows.

$$\mathrm{pH} = \mathrm{p}K_a + \log\frac{[un - ionized\ drug]}{[ionized\ drug]}$$

Therefore, the basic drug CIM can be expressed as in the following equation S2, substituting $[\mathrm{CIM}]_{mol}$ for the molecular type CIM, $[\mathrm{CIM}]_{ion}$ for the ionic type CIM and CIM-specific pKa for pKa.

$$\mathrm{pH} = \mathrm{p}K_a + \log\frac{[\mathrm{CIM}]_{mol}}{[\mathrm{CIM}]_{ion}}\ (\mathrm{S1})$$

Then, transforming equation S1, using pH, pKa, and $[\mathrm{CIM}]_{mol}$, $[\mathrm{CIM}]_{ion}$ can be expressed as in equation S2 below.

$$[\mathrm{CIM}]_{ion} = [\mathrm{CIM}]_{mol} \times 10^{\mathrm{p}K_a - \mathrm{pH}}(S2)$$

Therefore, the total concentration of CIM in the presence of 16 mM β-CD, $[\mathrm{CIM}]_t$, is the sum of $[\mathrm{CIM}]_{mol}$ for molecular CIM, $[\mathrm{CIM}]_{ion}$ for ionic CIM and [CIM/β-CD], which can be expressed as in Equation S3 below.

$$[\mathrm{CIM}]_{\mathrm{t}} = [\mathrm{CIM}]_{mol}\left(1 + 10^{\mathrm{p}K_a - \mathrm{pH}} + K[\beta\mathrm{CD}]\right)(\mathrm{S3})$$

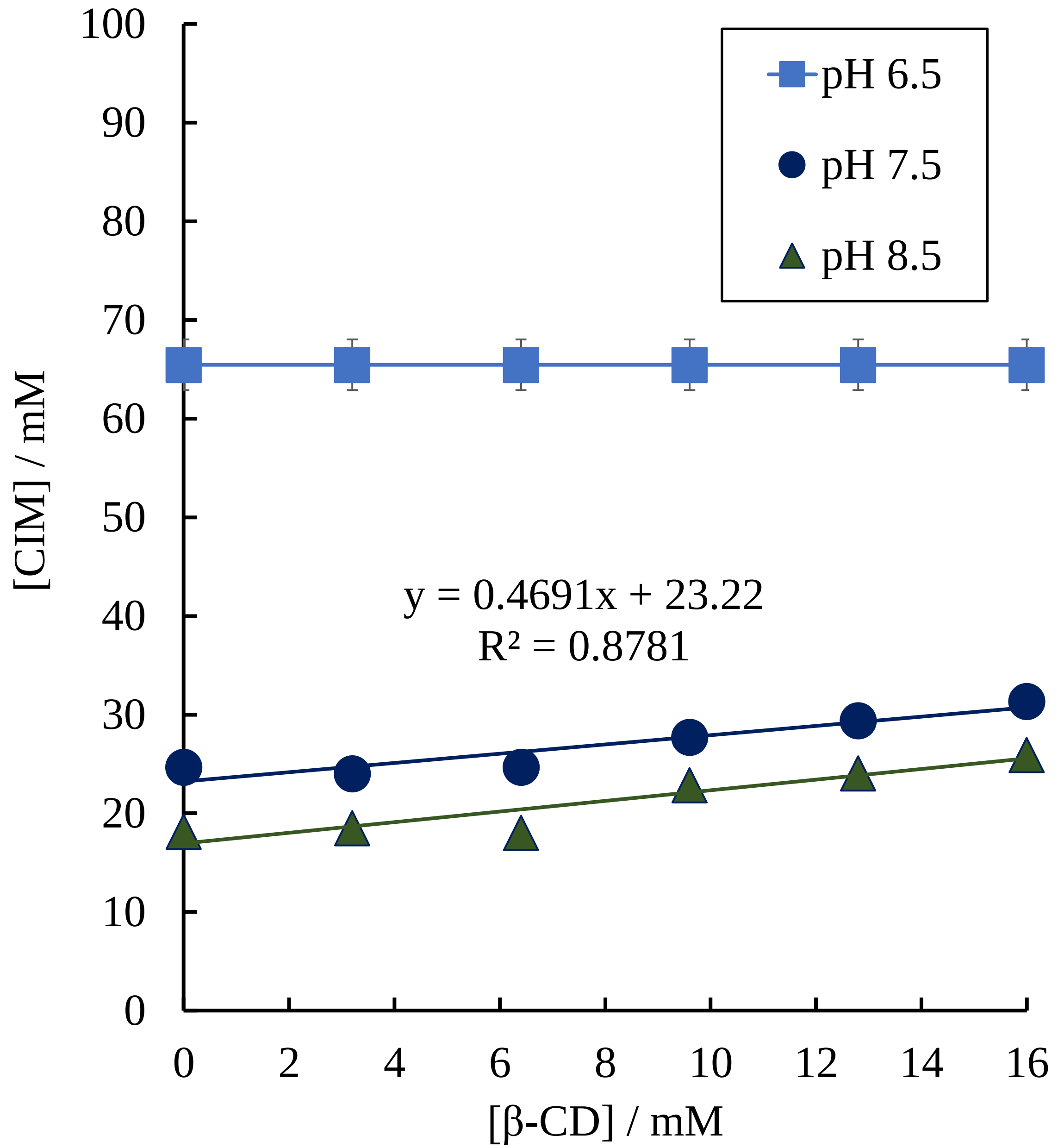

Figure S4. Solubility phase diagram of CIM. Solubility phase diagram (light blue closed squares) of CIM prepared in buffer of 100 mM phosphate buffer (pH 6.5); prepared in buffer of 100 mM phosphate buffer (pH 7.5) Solubility phase diagram of CIM (closed dark blue circles); solubility phase diagram of CIM prepared in a buffer of 100 mM phosphate buffer (pH 8.5) (closed dark green triangles).

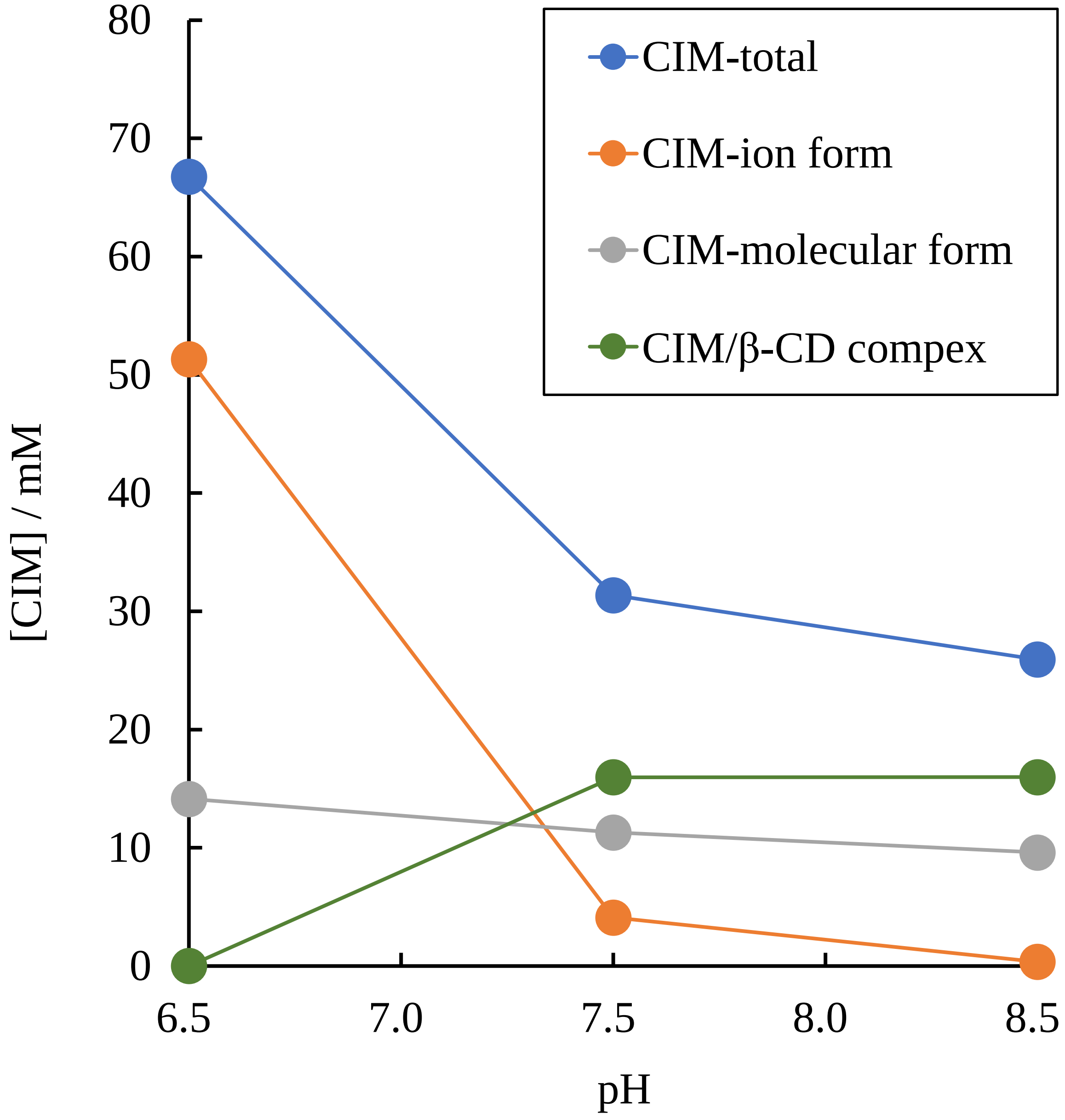

Figure S5. Concentrations of CIM in ionic form, CIM in molecular form, and CIM/β-CD in the presence of 16 mM β-CD in the solubility phase diagram of CIM at each pH calculated by the solver module of Microsoft Excel.

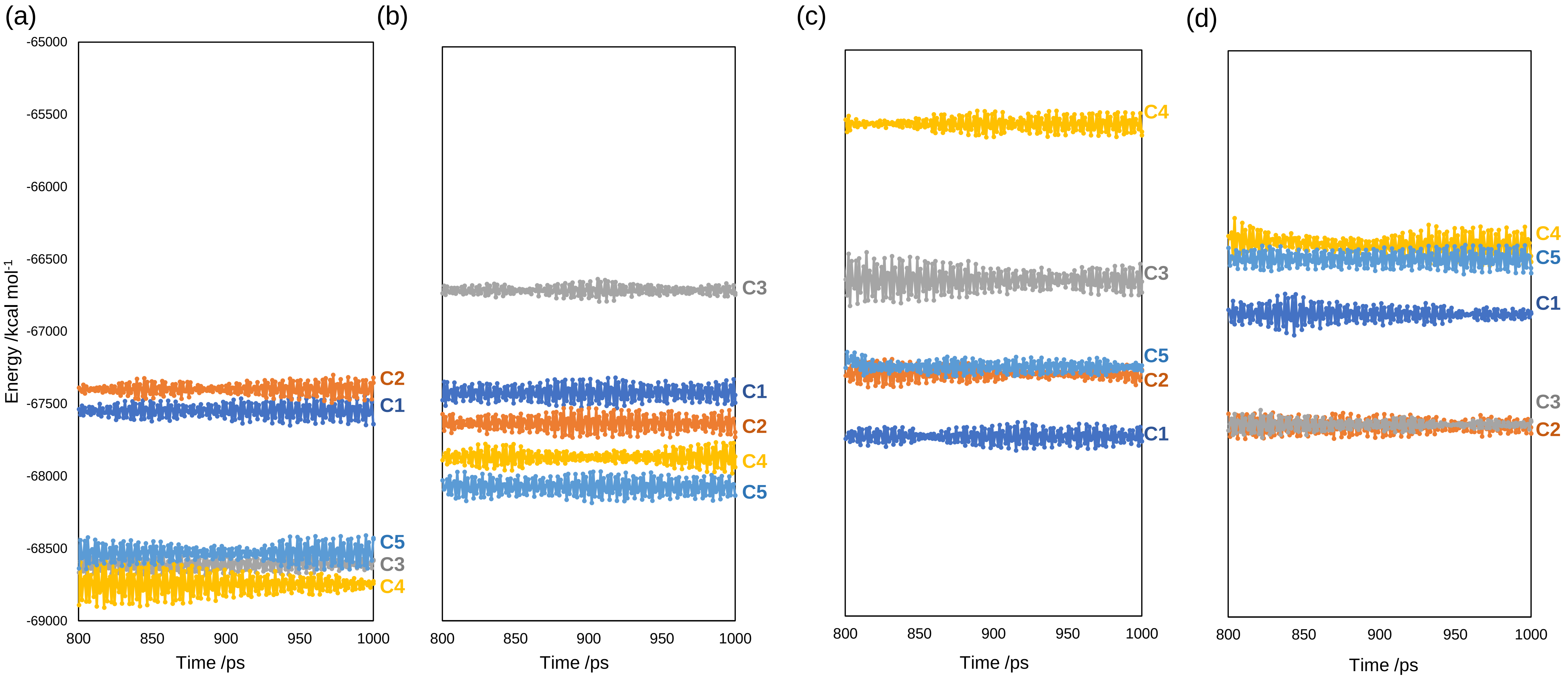

Figures S6. Molecular dynamic trajectory of neutral (a) DIC/FAM/β-CD complex, (b) DIC/CIM/HP-β-CD complex, (c) the IND/FAM/HP-β-CD complex, and (d) IND/CIM/HP-β-CD complex. C1; DIC or IND/β-CD complex and isolated FAM or CIM, C2; Inclusion of the thiol side of FAM or CIM/β-CD complex and isolated DIC or IND, C3; Inclusion of the five-membered ring side of FAM or CIM/β-CD complex and isolated DIC or IND, C4; Complexes of FAM or CIM (the thiol side) and DIC or IND in equal proximity to β-CD, C5; Complexes of FAM or CIM (the five-membered ring side) and DIC or IND in equal proximity to β-CD.

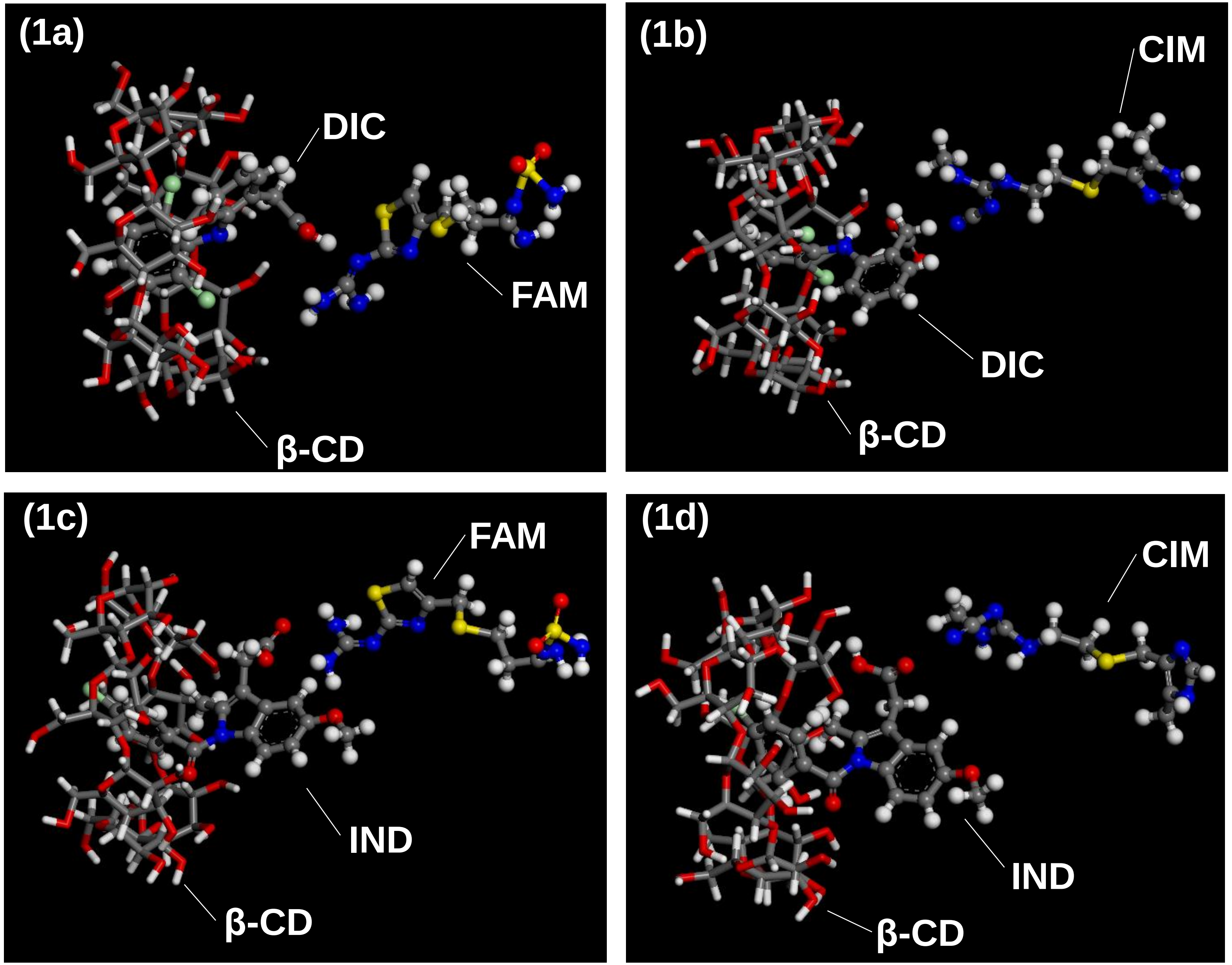

Figures S7 (1a-1d). Snap shots for the most stable structures of C1 complexes. (1a) the DIC/FAM/HP-β-CD complex, (1b) the DIC/CIM/HP-β-CD complex, (1c) the IND/FAM/HP-β-CD complex, and (1d) the IND/CIM/HP-β-CD complex. Images 1a-1d correspond to C1 (blue) of a-d in Fig. S6.

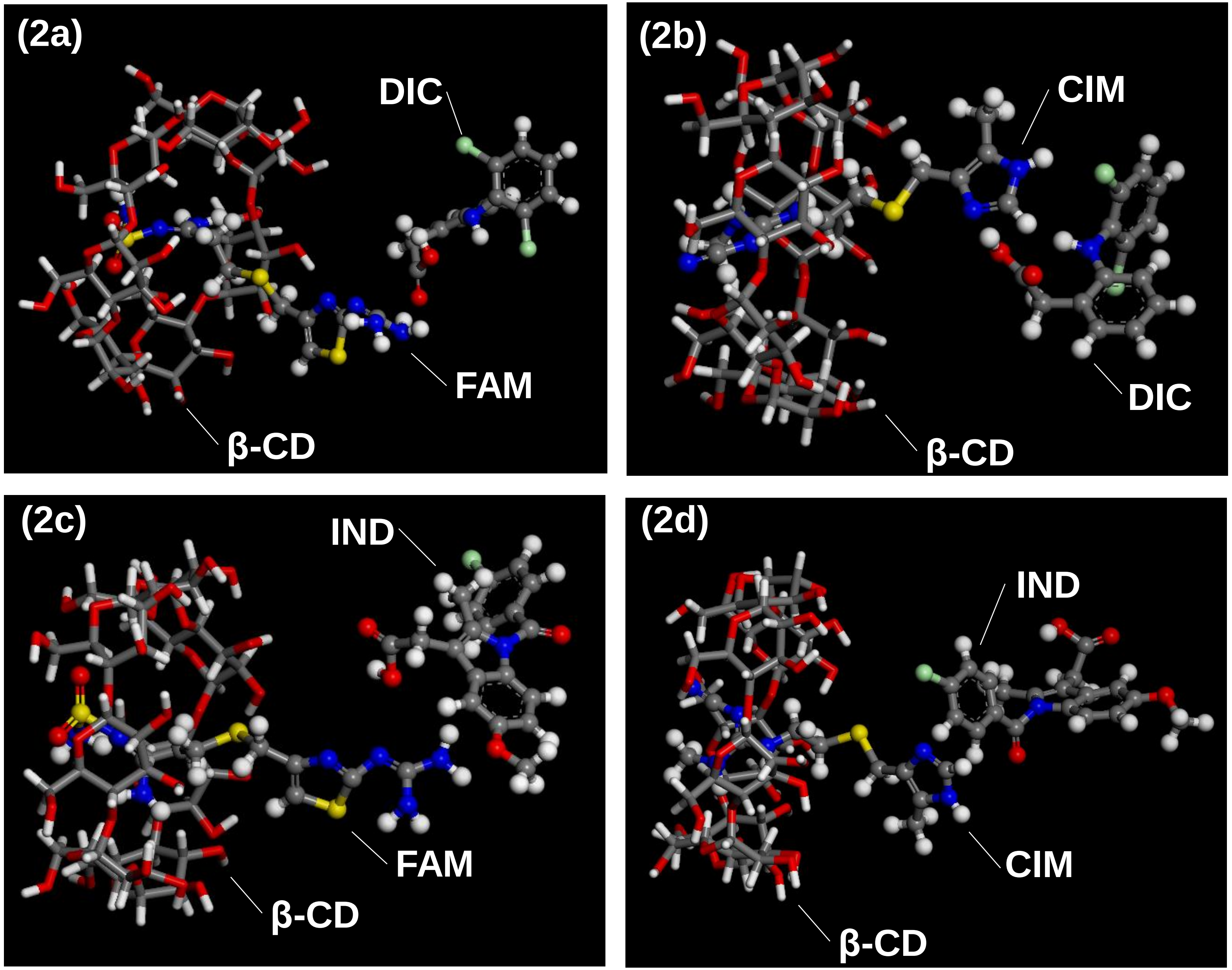

Figures S7 (2a-2d). Snap shots for the most stable structures of C2 complexes. (2a) the DIC/FAM/HP-β-CD complex, (2b) the DIC/CIM/HP-β-CD complex, (2c) the IND/FAM/HP-β-CD complex, and (2d) the IND/CIM/HP-β-CD complex. Images 2a-2d correspond to C2 (orange) of a-d in Fig. S6.

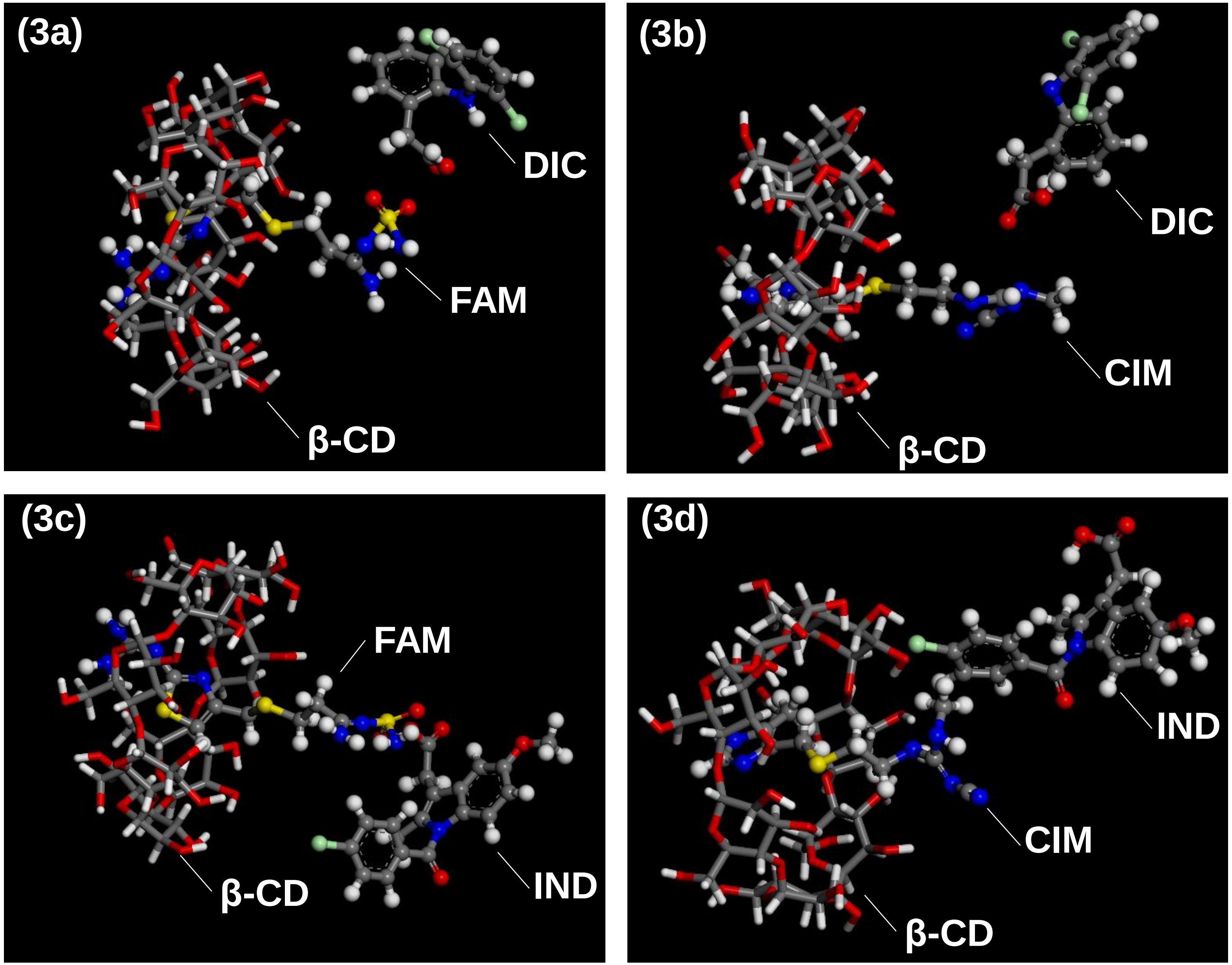

Figures S7 (3a-3d). Snap shots for the most stable structures of C3 complexes. (3a) the DIC/FAM/HP-β-CD complex, (3b) the DIC/CIM/HP-β-CD complex, (3c) the IND/FAM/HP-β-CD complex, and (3d) the IND/CIM/HP-β-CD complex. Images 3a-3d correspond to C3 (gray) of a-d in Fig. S6.

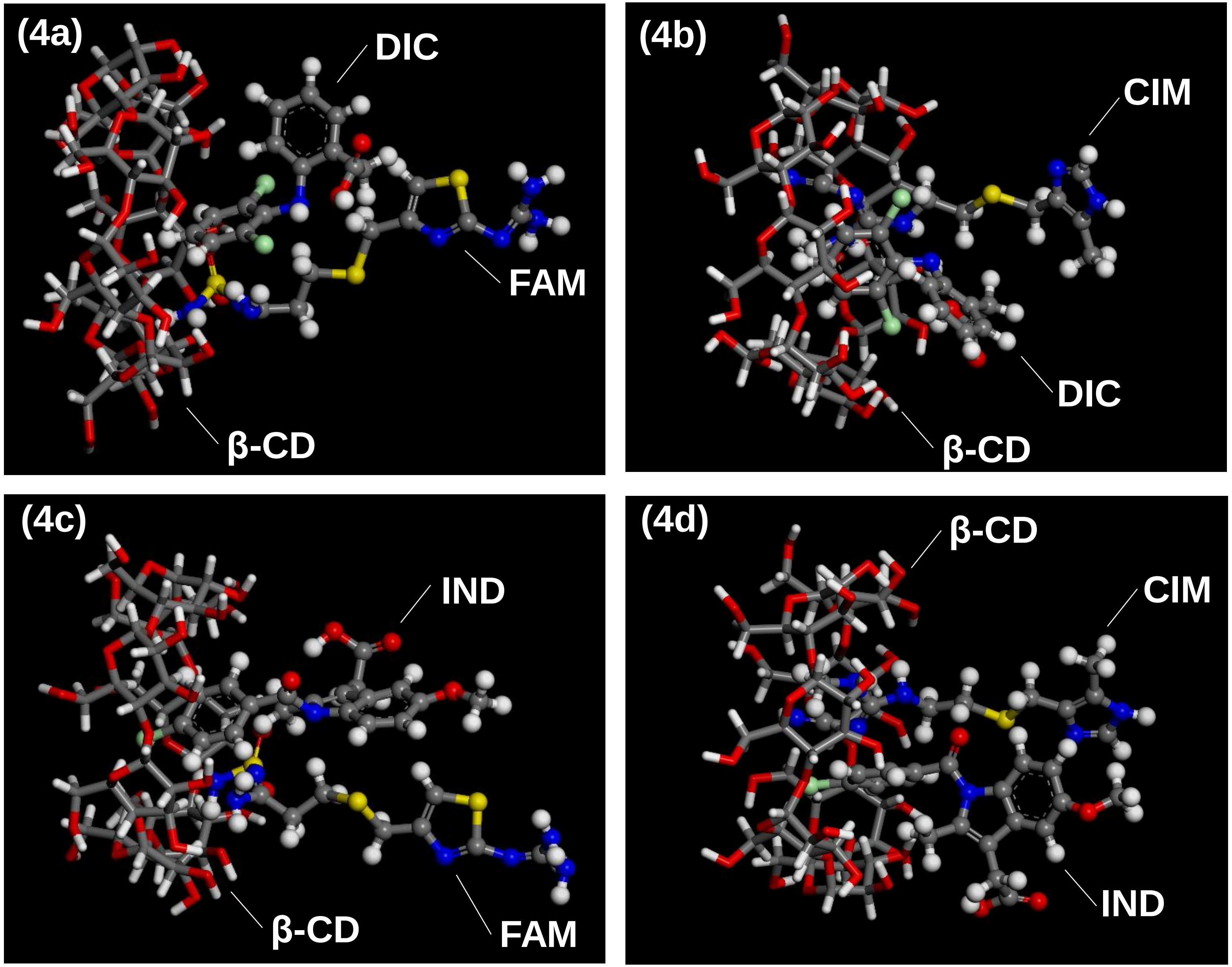

Figures S7 (4a-4d). Snap shots for the most stable structures of C4 complexes. (4a) the DIC/FAM/HP-β-CD complex, (4b) the DIC/CIM/HP-β-CD complex, (4c) the IND/FAM/HP-β-CD complex, and (4d) the IND/CIM/HP-β-CD complex. Images 4a-4d correspond to C4 (yellow) of a-d in Fig. S6.

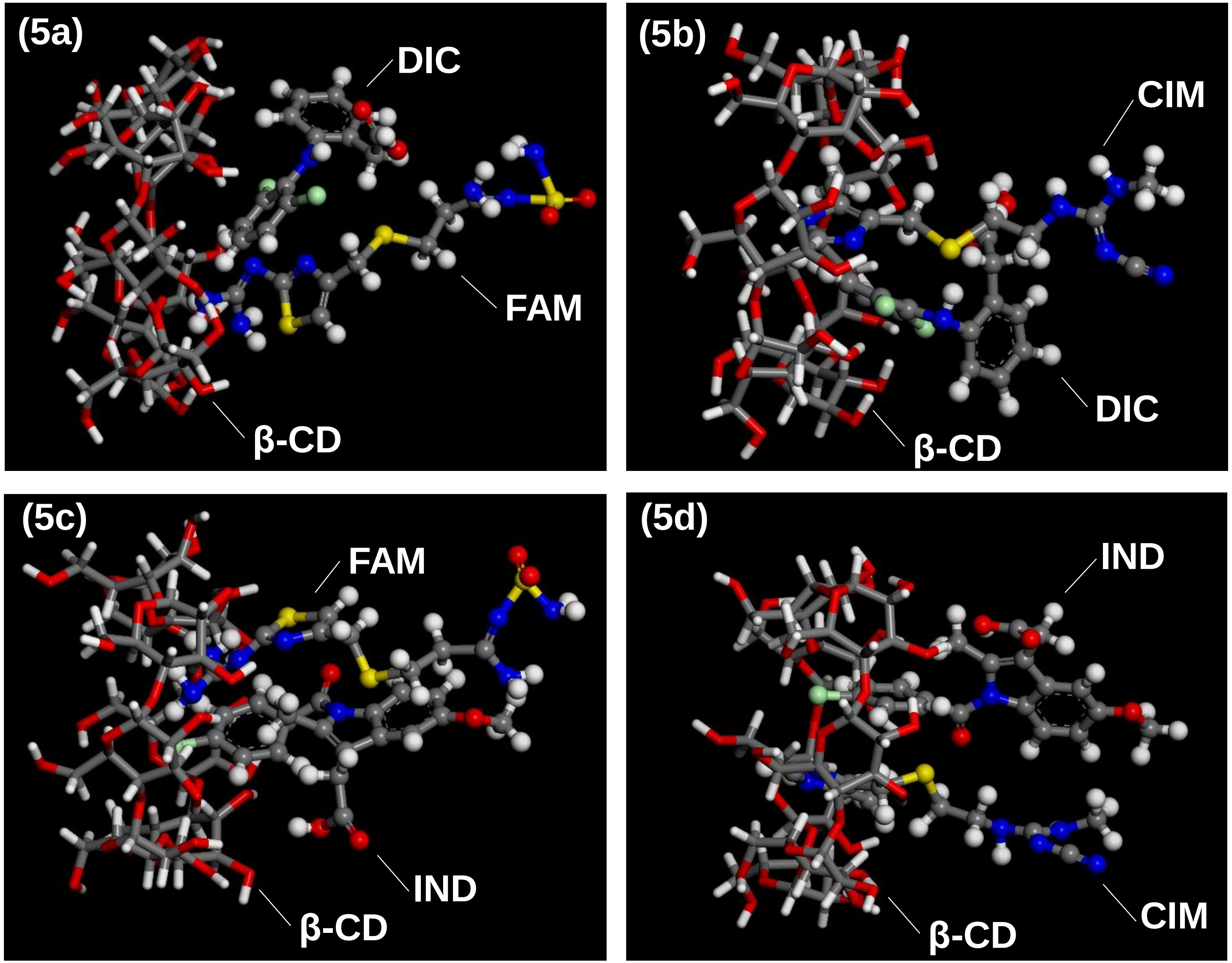

Figures S7 (5a-5d). Snap shots for the most stable structures of C5 complexes. (5a) the DIC/FAM/HP-β-CD complex, (5b) the DIC/CIM/HP-β-CD complex, (5c) the IND/FAM/HP-β-CD complex, and (5d) the IND/CIM/HP-β-CD complex. Images 5a-5d correspond to C5 (light blue) of a-d in Fig. S6.

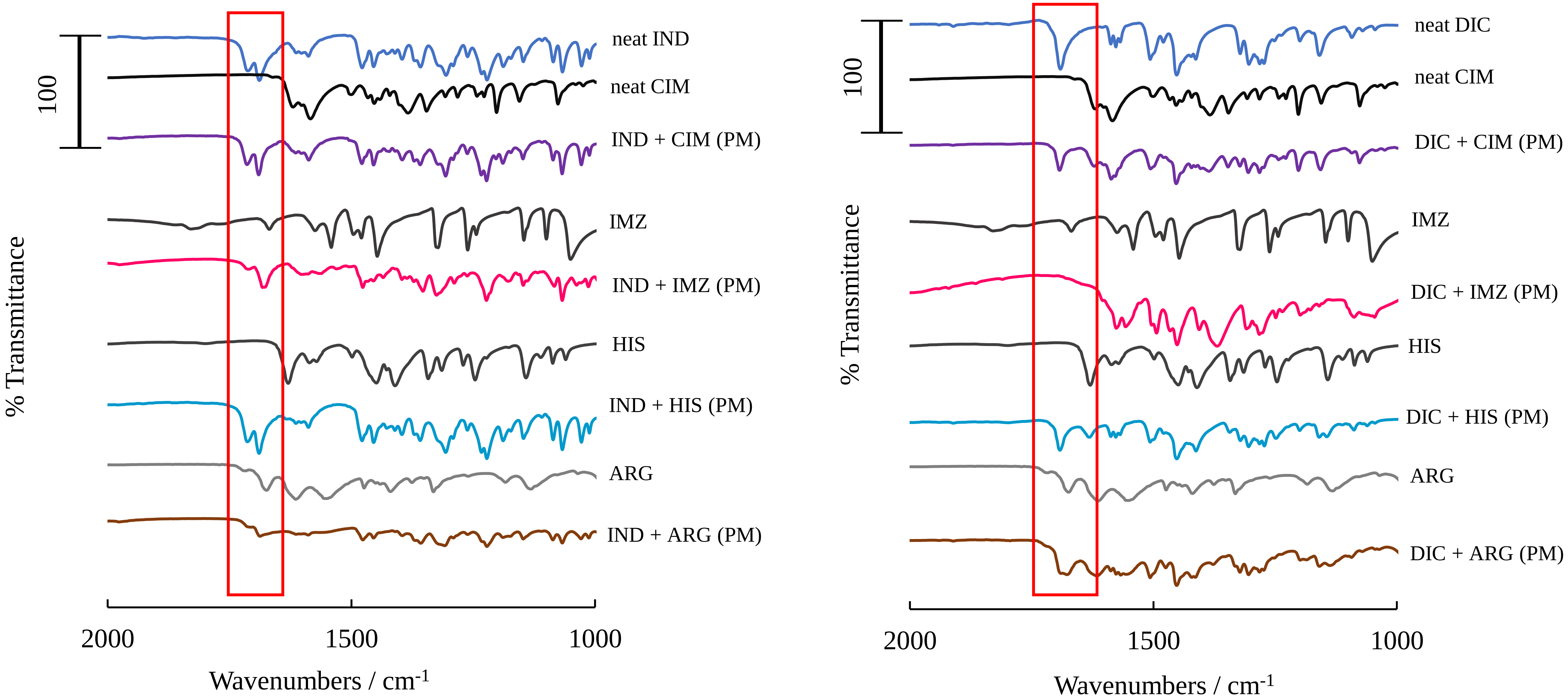

Figure S8. FTIR spectra of physical mixtures (PM) of basic and acidic drugs.

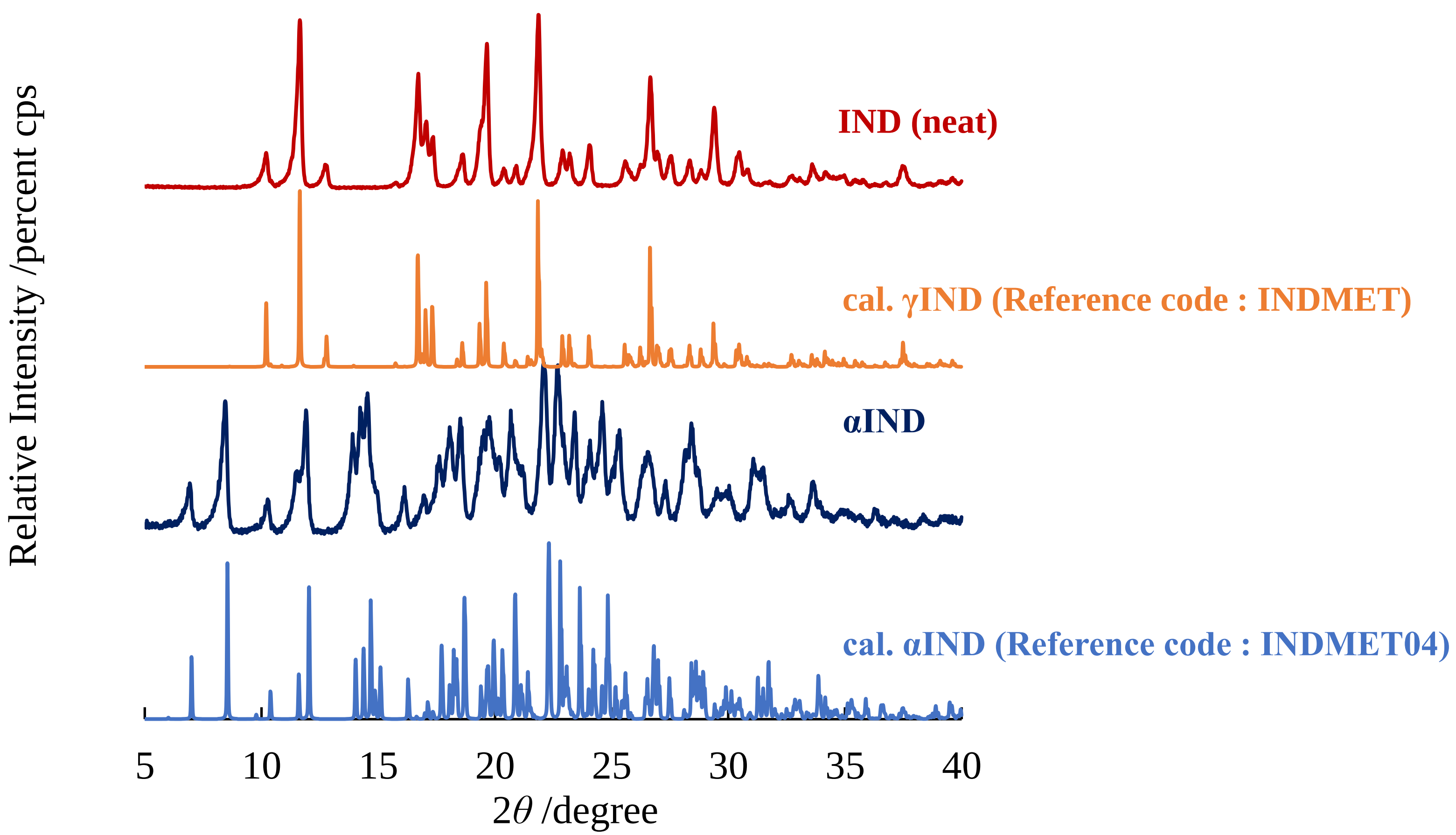

Figure S9. Diffractograms of γ-form IND and α-form IND by actual measurement and those of reported in the literature. The data can be obtained free of charge from The Cambridge Crystallographic Data Centre (CCDC) via www.ccdc.cam.ac.uk/getstructures.
